# Supplementary material for: Parental understanding of our communication of morbidity associated with paediatric cardiac surgery: a qualitative study
Source: BMJ Paediatr Open. 2020 Feb 26;4(1):e000578. doi: 10.1136/bmjpo-2019-000578 (PMC7047488; doi:10.1136/bmjpo-2019-000578)
Supplement: Author's manuscript [file bmjpo-2019-000578.draft_revisions.pdf]

BMJ Paediatrics Open

BMJ Paediatrics Open is committed to open peer review. As part of this commitment we make the peer review history of every article we publish publicly available.

When an article is published we post the peer reviewers' comments and the authors' responses online. We also post the versions of the paper that were used during peer review. These are the versions that the peer review comments apply to.

The versions of the paper that follow are the versions that were submitted during the peer review process. They are not the versions of record or the final published versions. They should not be cited or distributed as the published version of this manuscript.

BMJ Paediatrics Open is an open access journal and the full, final, typeset and author-corrected version of record of the manuscript is available on our site with no access controls, subscription charges or pay-per-view fees (http://bmjpaedsopen.bmj.com).

If you have any questions on BMJ Paediatrics Open's open peer review process please email info.bmjpo@bmj.com

BMJ Paediatrics Open

Parental understanding of our communication of morbidity associated with paediatric cardiac surgery

Journal:	*BMJ Paediatrics Open*
Manuscript ID	bmjpo-2019-000578
Article Type:	Original article
Date Submitted by the Author:	13-Sep-2019
Complete List of Authors:	Rajagopal, Veena; Great Ormond Street Hospital For Children NHS Foundation Trust,<br>Brown, Katherine; Great Ormond Street Hospital For Children NHS Foundation Trust<br>Pagel, Christina; University College London; Great Ormond Street Hospital For Children NHS Foundation Trust<br>Wray, Jo; Great Ormond Street Hospital For Children NHS Foundation Trust
Keywords:	Cardiac Surgery, Intensive Care, Qualitative research, Outcomes research, Psychology

SCHOLARONE™
Manuscripts

I, the Submitting Author has the right to grant and does grant on behalf of all authors of the Work (as defined in the below author licence), an exclusive licence and/or a non-exclusive licence for contributions from authors who are: i) UK Crown employees; ii) where BMJ has agreed a CC-BY licence shall apply, and/or iii) in accordance with the terms applicable for US Federal Government officers or employees acting as part of their official duties; on a worldwide, perpetual, irrevocable, royalty-free basis to BMJ Publishing Group Ltd ("BMJ") its licensees and where the relevant Journal is co-owned by BMJ to the co-owners of the Journal, to publish the Work in this journal and any other BMJ products and to exploit all rights, as set out in our licence.

The Submitting Author accepts and understands that any supply made under these terms is made by BMJ to the Submitting Author unless you are acting as an employee on behalf of your employer or a postgraduate student of an affiliated institution which is paying any applicable article publishing charge ("APC") for Open Access articles. Where the Submitting Author wishes to make the Work available on an Open Access basis (and intends to pay the relevant APC), the terms of reuse of such Open Access shall be governed by a Creative Commons licence – details of these licences and which Creative Commons licence will apply to this Work are set out in our licence referred to above.

Other than as permitted in any relevant BMJ Author's Self Archiving Policies, I confirm this Work has not been accepted for publication elsewhere, is not being considered for publication elsewhere and does not duplicate material already published. I confirm all authors consent to publication of this Work and authorise the granting of this licence.

Confidential: For Review Only

Parental understanding of our communication of morbidity associated with paediatric cardiac surgery

Veena Rajagopal [1], Katherine Brown [1], Christina Pagel [2], Jo Wray [1]

[1] Great Ormond Street Hospital Cardiac Unit London-United Kingdom

[2] University College London Clinical Operational Research Unit London-United Kingdom

Address for correspondence
Dr Jo Wray
Critical Care and Cardiorespiratory Directorate
Great Ormond Street Hospital
Great Ormond Street
London WC1N 3JH

Email: jo.wray@gosh.nhs.uk

Key words: parents, cardiac surgery, morbidity, communication

Confidential: For Review Only

Abstract

Background: As survival rates following paediatric cardiac surgery increase, it is important to focus on survivors' quality of life, which can be significantly impacted by morbidities associated with cardiac surgery. Parental understanding of the potential for postoperative morbidity is important for informed decision making. As part of a broader research study, we aimed to elicit parental understanding and experience of the communication of morbidities following their child's cardiac surgery, using traditional focus groups together with an online forum.

Methods: The Children's Heart Federation set up and moderated a closed, anonymous online discussion group via their Facebook page, focussing on morbidities, information needs and methods of providing families with information. Additionally, we ran three focus groups with parents/carers, moderated by an experienced independent professional. Focus groups were recorded and transcribed and a single transcript was generated from the online forum. All transcripts were thematically analysed.

Results: The forum ran over 3 months and involved 72 participants. The focus groups involved 13 participants. Three broad themes were identified: 1) Clinicians' use of language 2) Feeling unprepared for complications and 3) Information needs of families.

Conclusions: Clinicians' language is often misunderstood, with wide variability in the way morbidities are described, and between differing teams looking after the same child. Information may not be easily absorbed or retained by families, who often felt unprepared for morbidities that arose after their child's heart surgery. Here we propose key principles of good communication, tailored to the individual receiving it.

Confidential: For Review Only

Introduction

Short term mortality following paediatric cardiac surgery in the UK has fallen steadily over recent years (from 4.3% in 2000 to 2.4% in 2016). [1,2] As the large majority of children now survive into adulthood following heart surgery, it becomes increasingly important to consider any associated morbidity [3], its impact on quality of life and importantly, how information about morbidity is communicated to parents, care givers and patients.

Morbidity associated with paediatric cardiac surgery can be very challenging for patients and families. For those with the most severe morbidities the impact may be life changing or, in some cases, life-limiting. As more children with increasingly complex heart disease successfully undergo surgery, there are a growing number living with the impact of a range of morbidities arising as a consequence of surgery [4]. It is therefore imperative that information about morbidities is communicated in an empathic and clear way.

Very little is known about parental experience and understanding of our communication of morbidity associated with paediatric cardiac surgery. We do know from work done in other settings however [5,6] that levels of distress, time pressure and the quality of 'clinician-parent communication' can adversely affect parental understanding of information given, with the potential to limit parents' ability to make informed decisions. [7] Parents want their clinicians to be accessible, honest, caring, and use lay language at a pace that can be understood. [8]

In this paper we focus on family perceptions about the way healthcare professionals communicate regarding morbidities associated with paediatric cardiac surgery, which were

https://mc.manuscriptcentral.com/bmjpo

elicited as part of a wider study looking at the selection, definition, incidence and impact of morbidities following paediatric cardiac surgery. [4, 9,10]

Traditional methods of accessing parental views may exclude certain groups of people. [11,12] Logistics, time, and discomfort in face-to-face and group interaction may deter some individuals. As its use increases, the internet represents a key tool for the delivery and discussion of health care information. Online forums are an important way in which internet users seek this information and communicate with others who have similar health care experiences. There is an increasing focus on understanding the potential value of this method of data collection in qualitative research. [13,14] Forums have successfully been used in healthcare research, [15-20] providing a flexible and safe space, where open and honest discussions can be held over a period of months, in a conveniently non-synchronous way for potentially large numbers of users in different geographical areas. [11, 12, 21] There is no burden on travel, and participation results in minimal disruption to daily life.

We therefore hoped that including an online forum as a method of engaging with families, alongside more traditional approaches, might enable us to access the views of those who find it hard to attend in person or take part in interviews and focus groups, adopting a similar approach to that used by our group previously. [22] We report here views elicited from a charity-moderated online forum and from three focus groups.

Confidential: For Review Only

Methods

Design

A qualitative approach underpinned by an interpretivist framework, employing an online forum in addition to three more traditional focus groups was used as a method of data collection, to access participant viewpoints.

Participants and data collection

We report here the views of parents/carers of children with congenital heart disease who chose to participate in the study.

The Children's Heart Federation (CHF), a national parent charity, facilitated and moderated a closed, anonymous, online discussion group via their Facebook page, following a similar approach to that reported in a previous study.[22] The discussion group was advertised on the charity's home web page [23] to anyone who had experienced taking care of a child after heart surgery. Potential participants were directed to the charity's Facebook page where they could access information about the study and governance surrounding it. Anyone wishing to participate was asked to provide basic demographic details (age, gender, ethnicity, relationship to heart patient, geographical region) and on completion of this they were directed to the closed Facebook group, where they were able to respond to questions posted there. The research team provided questions to be posted on the forum at the start of the process and the charity were responsible for deciding when new questions should be posted or any prompts introduced, based on participant responses and the rate of responding. Questions posted on the forum are shown in Table 1. The forum took place over a three-month period.

Confidential: For Review Only

In addition, we held three focus groups, which took place in Glasgow, Birmingham, and London. These locations were chosen for their broad case mix and ethnic diversity. The focus groups were advertised via the CHF website (as for the online forum) and potential participants contacted the CHF if they wanted to take part. The CHF organised the groups, which were held on a Saturday and moderated by an independent, experienced researcher using as a framework the same questions that were used for the online forum (see table 1). Focus group participants provided written consent for their participation, recording and use of anonymised quotes in dissemination of the findings. Each focus group was audio-recorded and transcribed verbatim.

Ethical approval was granted by London City Road Research Ethics Committee (study number: 14-LO-1442).

Data management and analysis

Responses from the online forum were collated into a single transcript and anonymised prior to sending to the research team. The transcripts from the online forum and focus groups were thematically analysed (non -parent/carer responses were excluded from analysis). [24] Transcripts were read and codes attached to segments of data independently by members of the research team (JW/CP/VR). Similar codes were then merged to create themes. The researchers met to discuss the themes and agree the descriptive names assigned to them. Discussion continued until consensus was reached.

Patient and Public Involvement

Confidential: For Review Only

In the broader study, the list of morbidities linked to paediatric cardiac surgery was prioritised by a panel reflecting the views of professionals, parents and patients. This list was used in the questions posted on the forum and at the focus groups.

Participants in the online forum were unknown and not identifiable by the research team, therefore there was no involvement of participants in transcript review and coding.

This paper is motivated by families reporting inconsistencies in the communication of morbidity related to cardiac surgery.

Newsletters reporting progress over the course of the broader study were disseminated via the Children's Heart Federation.

Results

Demographics of participants

- ***Online forum***

The forum ran over 3 months and involved 72 participants (68 mothers, 1 father, 1 patient, 2 grandmothers; age 15-59 years). The vast majority of participants were white British (n=70; 97%) but there was a spread of participants across England, Wales and Scotland.

- ***Focus groups***

The three focus groups each lasted for approximately two hours and in total comprised 13 participants (10 mothers, 2 fathers, and 1 adult congenital heart disease patient). The ages of the parents' children ranged from 14 months - 24 years (only one 'child' was an adult, the others all being 14 years old or younger, with a median age of 5 years at the time of the

https://mc.manuscriptcentral.com/bmjpo

Confidential: For Review Only

group). The gender of the participants' children was 7 boys and 5 girls. Two children had recognised syndromes, and one of the mothers was bereaved.

Collated online forum and focus group data

A number of codes were identified from the online forum and focus group data, and collated to form three themes, with seven subthemes, relating to communication between healthcare professionals and families about morbidities arising from cardiac surgery. The themes are shown in Table 2 with illustrative quotes, and explained in more detail below.

Themes

Figure 1 illustrates the three themes with their related subthemes.

1. **Clinicians' use of language**

- **Comprehension**

Language used by clinicians about morbidities following surgery was often poorly understood by families. Jargon (e.g. words such as 'morbidity', 'stenotic', 'hypoplastic') was used instead of lay language (e.g. complications), and percentages were used to communicate risk of a morbidity occurring. These were often confusing to participants and led to misunderstanding.

Some parents felt it would have been useful to hear from another parent who had been through a similar experience before, someone who could tell them 'mum to mum' with 'no jargon', what to expect.

- **Consistency**

https://mc.manuscriptcentral.com/bmjpo

Confidential: For Review Only

Participants described different specialties as giving different messages about which morbidities might happen following surgery. Whilst some played down the chance of morbidities happening, others were seen as being more up-front during consent conversations. These inconsistencies between teams led to mistrust, and a perception that some clinicians were being more honest than others.

2. **Being unprepared for morbidities**

- **Differing priorities of health care professionals and families**

Parents often felt unprepared for morbidities when they arose. Differing priorities between clinical teams and families may have resulted in some morbidities not being discussed prior to surgery. For example, feeding difficulties, and the need for a nasogastric tube were of huge significance to families, however parents were frequently not made aware of this as a potential morbidity prior to surgery, perhaps because clinicians did not consider it with the same significance.

Another important example identified by many parents was the unanticipated psychological side effects on their child, the siblings and themselves, which were invariably lacking from discussions prior to cardiac surgery.

The lack of warning, and a consequent feeling of being completely unprepared to deal with these morbidities once they arose, was a clear difficulty expressed by parents.

- **Timing of consent**

Confidential: For Review Only

The timing of consent conversations was also highlighted as being suboptimal. Examples were given of consent being taken the night before surgery, when parents were feeling not only anxious, but also left with little time to reflect on or think through what had been said.

3. <u>Information needs of families</u>

- **There is no right amount of information**

It was clear that there was no 'right amount of information' that suits every individual. Whilst some parents wanted to know about all possibilities, others felt that too much information would have been overwhelming, potentially paralysing them in the decision-making process.

Participants felt that the detail of information being conveyed should be tailored according to the needs of the individual receiving it, rather than using a 'one size fits all' approach.

- **Types of information**

Written and audio-visual material were reported as helpful supplements. Being able to take something back to their accommodation, that could be read and digested in their own time, was valued. As well as more high-tech information, simple leaflets were appreciated as something that could be read without the need for internet access and a phone/computer.

- **Access to staff / lay support**

Parents valued access to a member of staff they could contact with further questions, and who could signpost them to other information as needed.

In addition, parents expressed that being able to speak to another parent, who had gone through something similar, and who *actually knew* how it felt to be a parent going through this, would have been a source of comfort and help.

Confidential: For Review Only

Discussion

Healthcare communication is a vital skill, influencing understanding of information and hence informed decision making. The importance of 'good communication' however goes beyond just understanding. Meert et al [8] ask whether the way in which clinicians communicate, may also impact on the psychological adjustment and functioning of parents and families.

Our study describes clinician, parental (predominantly maternal) and situational factors that may influence parental understanding of potential morbidities following paediatric cardiac surgery, and represent areas that can be targeted to improve this:

- Clinicians' use of jargon, the lack of consistency between clinicians and an individual clinician's skill in communication have been previously identified as important factors in patient comprehension and adherence [25,26] and in the current study all of these aspects impacted reported parental satisfaction and understanding of the conversation. Individual communication skills vary, with one participant noting that some clinicians are 'just better' at it than others. Though this may be an innate strength or weakness within an individual, clinicians need to reflect and improve upon their own communication skills, in the same way they would for any technical or procedural skills required of them. Observation of exemplar mentors, simulation training in communication including discussions with parents of children who have undergone cardiac surgery are useful strategies to improve these skills and gain parent centred perspectives. [27]

Confidential: For Review Only

- We know that the amount of information given by clinicians can have both positive and negative effects on parental anxiety. [7, 28] This was also reflected in our findings, suggesting that information given to parents needs to be tailored to the information needs of the individual receiving it. Taking the time to know what is right for the parent a clinician is speaking to, is an important investment not only in the parent's understanding, but also in their ability to make decisions and cope with their child's post-operative course. In addition, parents want to know what is likely to happen to *their* child, such that the risk of morbidities would ideally be tailored to the individual characteristics of the child for whom surgery is being discussed. However, as the population of cardiac children become increasingly complex, and as surgical and medical techniques evolve, tailoring assessments of risk is difficult and always subject to some uncertainty. How clinicians support parents and families in coping with uncertainty, needs to be an important part of specialist care.

- Stress and time pressure are barriers to informed consent. [29, 30] Participants commented on the difficulties they faced with consent the night before surgery. Where possible, a staged consent approach [31] should be used, such that an initial consultation will be used just to relay information, followed by a period of time allowing consideration by the parents, before a second conversation where consent is actually sought.

Many of our participants commented on the feeling of being 'unprepared'. We suggest future work should seek to understand if clinicians are able to mitigate some of the short and long-term distress experienced by families in this situation, through better

https://mc.manuscriptcentral.com/bmjpo

Confidential: For Review Only

communication and understanding of the parental perspective of morbidities and their impact on the child and family.

In this study, the online forum gave us access to many more parents than the focus groups (72 online, 13 at focus groups). This correlates with other reports suggesting the relative ease of online discussion compared to attending in person focus groups. [11,12,21]

Limitations

The data we obtained relied on participants' retrospective recall of conversations. Future work to look at clinician – parent communication in real time, would be a valuable insight into what is *actually* said, and the interpretation at the time - not only of parents, but also of clinicians: What did the parents understand from the conversation, and what did the clinicians believe the parents understood from the conversation. Furthermore, using an online forum to collect parent perceptions does not result in the same depth of information that would be possible in individual interviews and the approach precludes probing for further detail.

We recognise that both the online forum and the focus groups can in themselves exclude certain individuals due to lack of familiarity, language barriers, literacy barriers and lack of internet resources. In addition, our study lacked the views of males and non-Caucasians, who were under-represented in our participant sample, despite the chosen locations for the focus groups having a broad ethnic and socio-economic mix. The challenges of including ethnically, culturally and linguistically diverse populations, as well as fathers, in paediatric research studies are well documented. [32, 33] In order to capture these important often unheard views, we must find and adopt an innovative approach that successfully includes minority groups to ensure the broadest capture of parental/family views.

Confidential: For Review Only

Finally, we did not collect the same demographic information from participants in the online forum and the focus groups, which limits our ability to describe certain aspects of our participants across both data collection approaches.

Conclusions and suggestions for future practice

The nature of the online forum, and to a lesser extent the focus groups, promoted free debate and sharing of views with a large group of parents/carers, providing data that may not have been elicited in interviews.

The feedback we obtained suggests that there is wide variability and lack of consistency in the way clinicians describe complications, much of which may not be absorbed or retained by parents, particularly during times of extreme stress and distress. [27, 34,35]

Analysis of the data points towards the need for an individualised approach to communication around complications, based on 'high quality conversations', in which certain *key principles* of good communication are followed.

Future work to investigate the impact of 'good communication' on the short- and long-term psychological morbidity of parents and families would be a valuable next step, together with identifying how this may impact on physical and psychological outcomes in the children undergoing surgery.

In table 3 we list what we suggest are key principles for good communication.

Confidential: For Review Only

Funding details

This project was funded by the National Institute for Health Research Health Services and Delivery Research programme (Project No: 12/5005/06).

Acknowledgement

We would like to thank the Children's Heart Federation and Ms. Rehana Ahmed for their assistance in moderating the online forum and focus groups.

Research at Great Ormond Street Hospital is supported by the Great Ormond Street Hospital National Institute of Health Research Biomedical Research Centre.

Confidential: For Review Only

Contributorship statement

K Brown, C Pagel and J Wray planned the study. The forum and focus groups were conducted by R Ahmed and the Children's Heart Federation. V Rajagopal, C Pagel and J Wray analysed and reported results. V Rajagopal submitted the study.

What is already known about this topic:

- **Stress, time pressure and 'quality' of communication can adversely affect parental understanding, potentially limiting their ability to make informed decisions.**

What this study adds:

- **We have identified a range of clinician, parental and situational factors that may influence parental understanding during communication about morbidity following paediatric cardiac surgery.**
- **These factors represent targets for improvement and have led to the development of our principles of 'good communication', described in this paper.**

Confidential: For Review Only

References

1. Brown KL, Crowe S, Franklin R, McLean A, Cunningham D, Barron D, et al. Trends in 30-day mortality rate and case mix for paediatric cardiac surgery in the UK between 2000 and 2010. Open Heart. 2015;2(1):e000157. Epub 2015/04/22. doi: 10.1136/openhrt-2014-000157. PubMed PMID: 25893099; PubMed Central PMCID: PMCPMC4395835.

2. National Congential Heart Disease Annual Report 2013-2016. Available from: https://www.ucl.ac.uk/nicor/audits/congenital/documents/annual-reports/National_Congenital_Heart_Disease_2013_16_annual_report

3. Selection, definition and evaluation of important early morbidities associated with paediatric cardiac surgery. NIHR Evaluation TaS.Trials and Studies. [11 June 2018]. Available from: http://www.nets.nihr.ac.uk/projects/hsdr/12500506.

4. Brown KL, Ridout D, Pagel C, Wray J, Anderson D, Barron DJ, et al. Incidence and risk factors for important early morbidities associated with pediatric cardiac surgery in a UK population. J Thorac Cardiovasc Surg. 2019 Jun 12; DOI: 10.1016/j.jtcvs.2019.03.139

5. Sloper P. Predictors of distress in parents of children with cancer: a prospective study. J Pediatr Psychol. 2000;25(2):79-91. Epub 2000/05/23. PubMed PMID: 10820946.6.

6. Levi RB, Marsick R, Drotar D, Kodish ED. Diagnosis, disclosure, and informed consent: learning from parents of children with cancer. J Pediatr Hematol Oncol. 2000;22(1):3-12. Epub 2000/03/01. PubMed PMID: 10695815.

7. Miller VA, Drotar D, Burant C, Kodish E. Clinician-parent communication during informed consent for pediatric leukemia trials. J Pediatr Psychol. 2005;30(3):219-29. Epub 2005/03/24. doi: 10.1093/jpepsy/jsi032. PubMed PMID: 15784918.

8. Meert KL, Eggly S, Pollack M, Anand KJ, Zimmerman J, Carcillo J, et al. Parents' perspectives on physician-parent communication near the time of a child's death in the pediatric intensive care unit. Pediatr Crit Care Med. 2008;9(1):2-7. Epub 2008/05/15. doi: 10.1097/01.PCC.0000298644.13882.88. PubMed PMID: 18477906; PubMed Central PMCID: PMCPMC3198033.

9. Pagel C, Brown KL, McLeod I, Jepps H, Wray J, Chigaru L, et al. Selection by a panel of clinicians and family representatives of important early morbidities associated with paediatric cardiac surgery suitable for routine monitoring using the nominal group technique and a robust voting process. BMJ Open. 2017 May 1;7(5):e014743.

10. Brown KL, Pagel C, Brimmell R, Bull K, Davis P, Franklin RC, et al. Definition of important early morbidities related to paediatric cardiac surgery. Cardiol Young. 2017 May;27(4):747–56.

11. De Simoni A, Shanks A, Mant J, Skelton JR. Making sense of patients' internet forums: a systematic method using discourse analysis. Br J Gen Pract. 2014;64(620):e178-80. Epub 2014/02/26. doi: 10.3399/bjgp14X677671. PubMed PMID: 24567657; PubMed Central PMCID: PMCPMC3933853.

12. Balasooriya-Smeekens C, Bateman A, Mant J, De Simoni A. Barriers and facilitators to staying in work after stroke: insight from an online forum. BMJ Open. 2016;6(4):e009974. Epub 2016/04/08. doi: 10.1136/bmjopen-2015-009974. PubMed PMID: 27053267; PubMed Central PMCID: PMCPMC4823433.

13. Im EO, Chee W. An online forum as a qualitative research method: practical issues. Nurs Res. 2006;55(4):267-73. Epub 2006/07/20. PubMed PMID: 16849979; PubMed Central PMCID: PMCPMC2491331.

14. Im EO, Chee W. Practical guidelines for qualitative research using online forums. Comput Inform Nurs. 2012;30(11):604-11. Epub 2012/08/25. doi: 10.1097/NXN.0b013e318266cade. PubMed PMID: 22918135; PubMed Central PMCID: PMCPMC3727223.

15. Alinia H, Moradi Tuchayi S, Farhangian ME, Huang KE, Taylor SL, Kuo S, et al. Rosacea patients seeking advice: Qualitative analysis of patients' posts on a rosacea support forum. J

Confidential: For Review Only

Dermatolog Treat. 2016;27(2):99-102. Epub 2016/01/28. doi: 10.3109/09546634.2015.1133881. PubMed PMID: 26815357.
16. Jamison J, Sutton S, Mant J, De Simoni A. Online stroke forum as source of data for qualitative research: insights from a comparison with patients' interviews. BMJ Open. 2018;8(3):e020133. Epub 2018/04/01. doi: 10.1136/bmjopen-2017-020133. PubMed PMID: 29602848; PubMed Central PMCID: PMCPMC5884329.
17. Kaufman S, Whitehead KA. Producing, ratifying, and resisting support in an online support forum. Health (London). 2018;22(3):223-39. Epub 2016/02/07. doi: 10.1177/1363459315628043. PubMed PMID: 26851264.
18. Malik S, Coulson NS. The therapeutic potential of the internet: exploring self-help processes in an internet forum for young people with inflammatory bowel disease. Gastroenterol Nurs. 2011;34(6):439-48. Epub 2011/12/02. doi: 10.1097/SGA.0b013e318237a9ba. PubMed PMID: 22129797.
19. McKechnie V, Barker C, Stott J. The effectiveness of an Internet support forum for carers of people with dementia: a pre-post cohort study. J Med Internet Res. 2014;16(2):e68. Epub 2014/03/04. doi: 10.2196/jmir.3166. PubMed PMID: 24583789; PubMed Central PMCID: PMCPMC3961748.
20. Zhao J, Abrahamson K, Anderson JG, Ha S, Widdows R. Trust, empathy, social identity, and contribution of knowledge within patient online communities. Behaviour & Information Technology. 2013;32:1041-8. doi: 10.1080/0144929X.2013.819529.
21. Rhodes SD, Bowie DA, Hergenrather KC. Collecting behavioural data using the world wide web: considerations for researchers. J Epidemiol Community Health. 2003;57(1):68-73. Epub 2002/12/20. PubMed PMID: 12490652; PubMed Central PMCID: PMCPMC1732282.
22. Wray J, Brown K, Tregay J, Crowe S, Knowles R, Bull K, et al. Parents' Experiences of Caring for Their Child at the Time of Discharge After Cardiac Surgery and During the Postdischarge Period: Qualitative Study Using an Online Forum. J Med Internet Res. 2018;20(5):e155. Epub 2018/05/11. doi: 10.2196/jmir.9104. PubMed PMID: 29743157; PubMed Central PMCID: PMCPMC5966654.
23. http://www.chfed.org.uk/parents-wanted-for-a-study-looking-into-complications-following-heart-surgery/
24. Coffey A, Atkinson P. Making Sense of Qualitative Data:Complementary Research Strategies. Thousand Oaks, CA: Sage Publications; 1996.
25. Castro CM, Wilson C, Wang F, Schillinger D. Babel babble: physicians' use of unclarified medical jargon with patients. Am J Health Behav. 2007;31 Suppl 1:S85-95. Epub 2007/10/20. doi: 10.5555/ajhb.2007.31.supp.S85. PubMed PMID: 17931142.
26. Zolnierek KB, Dimatteo MR. Physician communication and patient adherence to treatment: a meta-analysis. Med Care. 2009;47(8):826-34. Epub 2009/07/09. doi: 10.1097/MLR.0b013e31819a5acc. PubMed PMID: 19584762; PubMed Central PMCID: PMCPMC2728700.
27. Levetown, M. Communicating With Children and Families: From Everyday Interactions to Skill in Conveying Distressing Information. Pediatrics. 2008; 121(5):e1441-60. DOI:10.1542/peds.2008-0565
28. Pomicino L, Maccacari E, Buchini S. Levels of anxiety in parents in the 24 hr before and after their child's surgery: A descriptive study. J Clin Nurs. 2018;27(1-2):278-87. Epub 2017/05/26. doi: 10.1111/jocn.13895. PubMed PMID: 28544343.
29. Barnlund DC. The mystification of meaning: doctor-patient encounters. J Med Educ. 1976;51(09):716-25. Epub 1976/09/01. PubMed PMID: 957387.
30. Janis I. Decision making under stress. In: Goldberger L, Breznitz S, editors. Handbook of stress : theoretical and clinical aspects. New York: The Free Press; 1993. p. 56-74.
31. Angiolillo AL, Simon C, Kodish E, Lange B, Noll RB, Ruccione K, et al. Staged informed consent for a randomized clinical trial in childhood leukemia: impact on the consent process. Pediatr Blood Cancer. 2004;42(5):433-7. Epub 2004/03/30. doi: 10.1002/pbc.20010. PubMed PMID: 15049015.

https://mc.manuscriptcentral.com/bmjpo

Confidential: For Review Only

32. Costigan CL, Cox MJ. Fathers' participation in family research: is there a self-selection bias? J Fam Psychol. 2001;15(4):706-20. Epub 2002/01/05. PubMed PMID: 11770476.
33. Williams A, Oulton K, Sell D, Wray J. Healthcare professional and interpreter perspectives on working with and caring for non-English speaking families in a tertiary paediatric healthcare setting. Ethn Health. 2017:1-14. Epub 2017/03/10. doi: 10.1080/13557858.2017.1294662. PubMed PMID: 28277020.
34. Scrimin S, Haynes M, Altoe G, Bornstein MH, Axia G. Anxiety and stress in mothers and fathers in the 24 h after their child's surgery. Child Care Health Dev. 2009;35(2):227-33. Epub 2009/02/21. doi: 10.1111/j.1365-2214.2008.00920.x. PubMed PMID: 19228156; PubMed Central PMCID: PMCPMC3496245.

35. Pagel C, Bull C, Utley M, Wray J, Barron DJ, Stoica S, et al. Exploring communication between parents and clinical teams following children's heart surgery: a survey in the UK. BMJ Paediatr Open. 2019;3(1):e000391

Confidential: For Review Only

Table 1: Questions asked on the online forum

**1. When thinking about children's heart surgery, what does the word "complication" mean to you?**
**2. When thinking about children's heart surgery, what does the word 'morbidity' mean to you? Can you give an example of how this affected you or your child?**
**3. As the experience of heart surgery becomes more distant and in the past, and your child is older, are there any difficulties that your child has now which you think may relate to the operation?**
**4. Thinking about yourself and your family, can you help us understand the impact that any complications had on you, your child or any other family members:**<br>➢ **While your child was still in hospital?**<br>➢ **After you got home?**
**6. We are thinking about measuring and recording how often complications happen, but we want to concentrate on the things that matter most to the child and family. Here is a list of problems that we know can happen:**<br>*• Children who have heart problems may sometimes experience different types of brain damage and this may lead to disability ranging from loss of hearing – to problems with learning or movement or all types of disability.*<br>*• A child may sometimes need an extra operation during the same stay in hospital that was not planned at the start.*<br>*• Infection may sometimes happen after heart surgery.*<br>*• A child's kidneys may stop working and need to be supported with a machine for a period of time.*<br>*• The muscle that helps a child to breathe may be weakened because the nerve supplying it is bruised or damaged – sometimes an operation is needed to strengthen the breathing muscle (diaphragm muscle).*<br>*• A child may need to stay in intensive care for a long time because he / she needed assistance with breathing or he / she needed tubes to remain in place.*<br>*• A child's gut may not digest milk or food and he / she may need to have nutrition via a drip.*<br>*• A child's heart may become very weak such that a machine to support the body called 'ECMO' was needed.*<br>*• Damage can happen to the nerve supply of the heart so that the child needs to have a pace maker put in to regulate the heart-beat.*<br>➢ **Which of these complications seems the worst or most worrying to you?**
**7. We are thinking about measuring and recording how often complications happen, but we want to concentrate on the things that matter most to the child and family. Here is a list of problems that we know can happen:**<br>*• Children who have heart problems may sometimes experience different types of brain damage and this may lead to disability ranging from loss of hearing – to problems with learning or movement or all types of disability.*

Confidential: For Review Only

• A child may sometimes need an extra operation during the same stay in hospital that was not planned at the start.<br>*• Infection may sometimes happen after heart surgery.*<br>*• A child's kidneys may stop working and need to be supported with a machine for a period of time.*<br>*• The muscle that helps a child to breathe may be weakened because the nerve supplying it is bruised or damaged – sometimes an operation is needed to strengthen the breathing muscle (diaphragm muscle).*<br>*• A child may need to stay in intensive care for a long time because he / she needed assistance with breathing or he / she needed tubes to remain in place.*<br>*• A child's gut may not digest milk or food and he / she may need to have nutrition via a drip.*<br>*• A child's heart may become very weak such that a machine to support the body called 'ECMO' was needed.*<br>*• Damage can happen to the nerve supply of the heart so that the child needs to have a pace maker put in to regulate the heart-beat.*<br>➢ **Could you please tell us why you think one or more of these complications is 'worse' than other complications?**<br>➢ **Are there things that happened to your child after their operation that you think 'went wrong' which should be on this list but isn't?**
**8. Could you please let us know what information you were provided with regarding the complications of your child's heart surgery?**
**9. (a) Do you feel you were provided with the right amount of information regarding complications before surgery?**<br>**9. (b) How much information would you like (more or less)? How much detail is helpful?**
**10. What visual (books etc) methods could be developed for letting families know the risk of complications to better inform them about what might happen after surgery?**
**11. What do you think about everyone being able to see (on the internet) the numbers of complications occurring after children's heart surgery at different hospitals?**
**12. (a) Do you have any further comments regarding complications following heart surgery? Is there anything we should know to improve services? Is there anything else you can think of?**<br>**12. (b) Is there anything we could have done to improve the online study? Do you think we should have asked questions more frequently, for example? Any other suggestions?**

Confidential: For Review Only

Table 2: Themes and sub-themes

Themes	Sub-themes	Quotes
**1.Clinicians' use of language**	***Comprehension***	▪ *"It's really easy to get yourself tied up in knots with risks and percentages, when they're really not explained properly"*<br>▪ *"Percentages are rarely accurate anyway, so why do we - as parents - hang on to them so dearly (me included!!!!). We were given 13% but it wasn't properly explained that this was risks (i.e. morbidity) not death alone."*<br>▪ *"I think it's assumed that parents have a basic understanding of statistics when it's such a complicated measurement and it's not properly explained to us."*<br>▪ *"It's just jargon...Please...English...so we can understand it!"*<br>▪ *"...some doctors are better than others...at explaining"*<br>▪ *"they go into the usual doctor terminology" "they use hypoplastic instead of small, stenotic instead of stiff"*<br>▪ *"Morbidity to me means death."*<br>▪ *'Just to talk mum to mum...then you're not getting the medical jargon'*
	Consistency	▪ *'I found a huge discrepancy between the way the cardiologists describe surgery i.e. very optimistic, complications are rare, and the surgeons who spell it out in order to cover themselves. Personally, I prefer the latter as it means when it does happen you are aware of it and know it has happened before, whereas the former makes you feel so unlucky and wondering why things have happened'.*<br>▪ *"We were given very detailed information by the surgeons on the eve of our sons' op but ... up until that time we had only seen cardiologists who were really quite blasé...We were told 'he'll be fine' they do switches all the time. Turns out it was far from that..."*
**2.Being unprepared for complications**	***Differing priorities of Health Care Professionals (HCP) and families***	▪ *"NG feeding was never something I thought about when we considered the prospect of having a congenital heart disease child."*<br>▪ *"We weren't mentally prepared for the longer stay as we were told 'in and out in 5 days'."*<br>▪ *"I really wish someone had prepared me for the psychological side effects... anything explaining how trauma and complications can have a negative impact on your child's self-esteem and mental well-being"*<br>▪ *"Tell parents beforehand...This is very likely going to affect development in growth, height, learning and development and things like that"*<br>▪ *"They do tell you some of the physical things that might happen" but not "how it might affect...a person's behaviour or emotions"*

Confidential: For Review Only

	Timing of consent	▪ *"You know when they do the consent forms it's usually the night before surgery which...is a bad idea, because you're not taking that in... if you did it a week before...you take in a lot more... It's easy for you to digest and understand"*
**3.Information needs of families**	***No right amount of information***	▪ *"Some parents will want to know everything and others want to know as little as possible"*<br>▪ *"If we knew all the potential outcomes I think signing the consent would have been so much harder"*<br>▪ *"You can't have a blanket rule of 'We must tell them every possible thing that could go wrong' or, 'We only tell them the most common'. You need to look at it case-by-case"*<br>▪ *"It's such a delicate balance…you've got to try and test the waters with the patient's parents and the patient's themselves to find out how much information...you need to give this person. It has to be an individual case-by-case scenario"*
	Types of information	▪ *"Something to take away and look at and digest in your own time"*<br>▪ *"Even a simple information sheet…you might not have internet access…you might need to go into your room or sit by the bed and have another look at it a bit later"*<br>▪ *"Little Hearts Matter...DVD pack for antenatal diagnosis. It was brilliant."*
	**Access to staff / lay support**	▪ *"Community Liaison Nurses are very useful and parents should be given contact numbers as a matter of course "*<br>▪ *"knowing who to ask…that there's somebody you can ask about things you might spot…signposting"*

Confidential: For Review Only

Table 3: Principles of good communication

1.	Openness
2.	Avoid jargon
3.	Include emotional and developmental complications during consent
4.	Consistent communication within and across all teams
5.	Appropriate timing of conversations
6.	Tailor conversations to the individual in front of you
7.	Individualise risk – what is likely for *this* particular child?
8.	Provide written and audio-visual material
9.	Provide a liaison link professional with contacts to parental support groups

Confidential: For Review Only

Confidential: For Review Only

Figure 1: Summary of feedback from parents about how complications were communicated to them by healthcare professionals

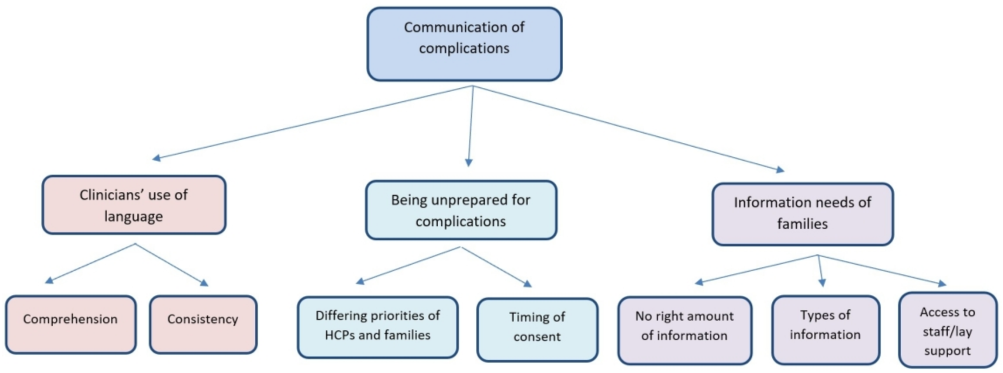

527x240mm (96 x 96 DPI)

BMJ Paediatrics Open

Parental understanding of our communication of morbidity associated with paediatric cardiac surgery: a qualitative study.

Journal:	*BMJ Paediatrics Open*
Manuscript ID	bmjpo-2019-000578.R1
Article Type:	Original article
Date Submitted by the Author:	13-Nov-2019
Complete List of Authors:	Rajagopal, Veena; Great Ormond Street Hospital For Children NHS Foundation Trust,<br>Brown, Katherine; Great Ormond Street Hospital For Children NHS Foundation Trust<br>Pagel, Christina; University College London; Great Ormond Street Hospital For Children NHS Foundation Trust<br>Wray, Jo; Great Ormond Street Hospital For Children NHS Foundation Trust
Keywords:	Cardiac Surgery, Intensive Care, Qualitative research, Outcomes research, Psychology

SCHOLARONE™
Manuscripts

I, the Submitting Author has the right to grant and does grant on behalf of all authors of the Work (as defined in the below author licence), an exclusive licence and/or a non-exclusive licence for contributions from authors who are: i) UK Crown employees; ii) where BMJ has agreed a CC-BY licence shall apply, and/or iii) in accordance with the terms applicable for US Federal Government officers or employees acting as part of their official duties; on a worldwide, perpetual, irrevocable, royalty-free basis to BMJ Publishing Group Ltd ("BMJ") its licensees and where the relevant Journal is co-owned by BMJ to the co-owners of the Journal, to publish the Work in this journal and any other BMJ products and to exploit all rights, as set out in our licence.

The Submitting Author accepts and understands that any supply made under these terms is made by BMJ to the Submitting Author unless you are acting as an employee on behalf of your employer or a postgraduate student of an affiliated institution which is paying any applicable article publishing charge ("APC") for Open Access articles. Where the Submitting Author wishes to make the Work available on an Open Access basis (and intends to pay the relevant APC), the terms of reuse of such Open Access shall be governed by a Creative Commons licence – details of these licences and which Creative Commons licence will apply to this Work are set out in our licence referred to above.

Other than as permitted in any relevant BMJ Author's Self Archiving Policies, I confirm this Work has not been accepted for publication elsewhere, is not being considered for publication elsewhere and does not duplicate material already published. I confirm all authors consent to publication of this Work and authorise the granting of this licence.

Confidential: For Review Only

Parental understanding of our communication of morbidity associated with paediatric cardiac surgery: a qualitative study

Veena Rajagopal [1], Katherine Brown [1], Christina Pagel [2], Jo Wray [1]

[1] Heart and Lung, Great Ormond Street Hospital for Children NHS Foundation Trust, London, United Kingdom

[2] Clinical Operational Research Unit, University College London, United Kingdom

Address for correspondence
Dr Jo Wray
Critical Care and Cardiorespiratory Directorate
Great Ormond Street Hospital
Great Ormond Street
London WC1N 3JH

Email: jo.wray@gosh.nhs.uk

Key words: parents, cardiac surgery, morbidity, communication

Confidential: For Review Only

Abstract

Background: Following paediatric cardiac surgery, quality of life may be significantly impacted by morbidities associated with cardiac surgery. Parental understanding of the potential for postoperative morbidity is important for informed decision making. As part of a broader research study, we aimed to elicit parental understanding and experience of the communication of morbidities following their child's cardiac surgery, using traditional focus groups together with an online forum.

Methods: The Children's Heart Federation set up and moderated a closed, anonymous online discussion group via their Facebook page, focussing on complications, information needs and methods of providing families with information. Additionally, we ran three focus groups with parents/carers, moderated by an experienced independent professional. Focus groups were recorded and transcribed and a single transcript was generated from the online forum. All transcripts were thematically analysed.

Results: All data were collected in 2014. The forum ran over 3 months in 2014 and involved 72 participants. Focus groups involved 13 participants. Three broad themes were identified: 1) Clinicians' use of language 2) Feeling unprepared for complications and 3) Information needs of families.

Conclusions: Clinicians' language is often misunderstood, with wide variability in the way morbidities are described, and between differing teams looking after the same child. Information may not be easily absorbed or retained by families, who often felt unprepared for morbidities that arose after their child's heart surgery. Here we propose key principles of good communication tailored to the individual receiving it.

Confidential: For Review Only

Introduction

Short term mortality following paediatric cardiac surgery in the UK has fallen steadily over recent years (from 4.3% in 2000 to 2.4% in 2016). [1, 2] As the large majority of children now survive into adulthood following heart surgery, it becomes increasingly important to consider any associated morbidities, as described previously, [3] their impact on quality of life and importantly, how information about morbidity is communicated to parents, care givers and patients.

Morbidity associated with paediatric cardiac surgery can be very challenging for patients and families. For those with the most severe morbidities the impact may be life changing or, in some cases, life-limiting. As more children with increasingly complex heart disease successfully undergo surgery, there are a growing number living with the impact of a range of morbidities arising as a consequence of surgery.[4] It is therefore imperative that information about morbidities is communicated in an empathic and clear way.

Very little is known about parental experience and understanding of our communication of morbidity associated with paediatric cardiac surgery. We do know from work done in other settings, however [5, 6], that levels of distress, time pressure and the quality of 'clinician-parent communication' can adversely affect parental understanding of information given, with the potential to limit parents' ability to make informed decisions. [7] Parents want their clinicians to be accessible, honest, caring, and use lay language at a pace that can be understood. [8]

https://mc.manuscriptcentral.com/bmjpo

Confidential: For Review Only

In this paper we focus on family perceptions about the way healthcare professionals communicate regarding morbidities associated with paediatric cardiac surgery, which were elicited as part of a wider study looking at the selection, definition, incidence and impact of morbidities following paediatric cardiac surgery. [3, 4, 9, 10]

Traditional methods of accessing parental views may exclude certain ‘hard to reach’ groups of people, including those who are more geographically remote and those from culturally and ethnically diverse backgrounds. [11, 12] Logistics, time, and discomfort in face-to-face and group interaction may deter some individuals. As its use increases, the internet represents a key tool for the delivery of health care information. Online forums are an important way in which internet users seek this information and communicate with others who have similar health care experiences. There is an increasing focus on understanding the potential value of this method of data collection in qualitative research. [13, 14] Forums have successfully been used in healthcare research, [15-20] providing a flexible and safe space, where open and honest discussions can be held over a period of months, in a conveniently non-synchronous way for potentially large numbers of users in different geographical areas. [11, 12, 21] There is no burden on travel, and participation results in minimal disruption to daily life.

We therefore hoped that including an online forum as a method of engaging with families, alongside more traditional approaches, might enable us to access the views of those who find it hard to attend in person or take part in interviews and focus groups, adopting a similar approach to that used by our group previously. [22] We report here views elicited from a charity-moderated online forum and from three focus groups.

Confidential: For Review Only

Methods

Design

A qualitative approach underpinned by an interpretivist framework, employing an online forum in addition to three more traditional focus groups was used as a method of data collection, to access participant viewpoints.

Participants and data collection

The Children's Heart Federation (CHF), a national parent charity, facilitated and moderated a closed, anonymous, online discussion group via their Facebook page, following a similar approach to that reported in a previous separate study.[22] The discussion group was advertised on the charity's home web page [23] and anyone who had experienced taking care of a child after heart surgery was eligible to participate. Potential participants were directed to the charity's Facebook page where they could access information about the study and governance surrounding it. Anyone wishing to participate was asked to provide basic demographic details (age, gender, ethnicity and geographical region) and on completion of this they were directed to the closed Facebook group, where they were able to respond to questions posted there. The research team provided questions to be posted on the forum at the start of the process and the charity were responsible for deciding when new questions should be posted or any prompts introduced, based on participant responses and the rate of responding. New questions were introduced when no new information was being posted – i.e. when data saturation had been reached for each question. Questions

Confidential: For Review Only

posted on the forum are shown in Table 1. The forum took place over a three-month period in 2014.

In addition, we held three focus groups, which took place in Glasgow, Birmingham, and London. This additional method of participation was included to enable those who wanted to participate via a more traditional face-to-face approach to do so. These locations were chosen for their broad case mix and ethnic diversity. The focus groups were advertised via the CHF website (as for the online forum) [23] and potential participants contacted the CHF if they wanted to take part. The CHF organised the groups, which were held on a Saturday and were moderated by an independent, experienced researcher using as a framework the same questions that were used for the online forum (see table 1). Focus group participants provided written consent for their participation, recording and use of anonymised quotes in dissemination of the findings. Each focus group was audio-recorded and transcribed verbatim.

Ethical approval was granted by London City Road Research Ethics Committee (study number: 14-LO-1442).

Data management and analysis

Responses from the online forum were collated into a single transcript. The transcripts from the online forum and focus groups were thematically analysed, [24] enabling identification, analysis and reporting of patterns within the data [25] related to parental understanding and experience of the communication of morbidities following their child's

Confidential: For Review Only

cardiac surgery. Non-parent/carer responses were excluded from analysis. Transcripts were read and codes attached to segments of data independently by members of the research team (JW/CP/VR). Similar codes were then merged to create themes. The researchers met to discuss the themes and agree the descriptive names assigned to them. Discussion continued until consensus was reached.

Participants in the online forum were unknown and not identifiable by the research team, therefore there was no involvement of participants in transcript review and coding.

Patient and Public Involvement

In the broader study, the list of morbidities linked to paediatric cardiac surgery was prioritised by a panel reflecting the views of professionals, parents and patients. This list was used in the questions posted on the forum and at the focus groups.

The reporting of these data is motivated by families reporting inconsistencies in the communication of morbidity related to cardiac surgery.

Results

Demographics of participants

- ***Online forum***

The forum ran over 3 months in 2014 and involved 72 participants (68 mothers, 1 father, 1 patient, 2 grandmothers; age 15-59 years). The vast majority of participants were white

Confidential: For Review Only

British (n=70; 97%) but there was a spread of participants across England, Wales and Scotland.

- ***Focus groups***

The three focus groups took place in 2014, each lasting approximately two hours and in total comprised 13 participants (10 mothers, 2 fathers, and 1 adult congenital heart disease patient). The ages of the parents' children ranged from 14 months - 24 years (however only one 'child' was an adult, the others all being 14 years old or younger, with a median age of 5 years at the time of the group). The gender of the participants' children was 7 boys and 5 girls. Two children had recognised syndromes, and one of the mothers was bereaved.

Collated online forum and focus group data

A number of codes were identified from the online forum and focus group data, and collated to form three themes, with seven subthemes, relating to communication between healthcare professionals and families about complications arising from cardiac surgery. The themes are shown in Table 2, with illustrative quotes, and explained in more detail below.

Themes

Figure 1 illustrates the three themes with their related subthemes.

1.Clinicians' use of language

- **Comprehension**

Confidential: For Review Only

Language used by clinicians about complications of surgery was often poorly understood by families. Jargon (e.g. words such as 'morbidity', 'stenotic', 'hypoplastic') was used instead of lay language, and percentages were used to communicate risk. These were often confusing to participants and led to misunderstanding.

Some parents felt it would have been useful to hear from another parent who had been through a similar experience before, someone who could tell them 'mum to mum' with 'no jargon', what to expect.

- **Consistency**

Participants described different specialties as giving different messages about which morbidities might happen following surgery. Whilst some played down the chance of morbidities happening, others were seen as being more up-front during consent conversations. These inconsistencies between teams led to mistrust, and a perception that some clinicians were being more honest than others.

2.Being unprepared for complications

- **Differing priorities of health care professionals and families**

Parents often felt unprepared for morbidities when they arose. Differing priorities between clinical teams and families may have resulted in some morbidities not being discussed prior to surgery. For example, feeding difficulties, and the need for a nasogastric tube were of huge significance to families, however parents were frequently not made aware of this as a potential morbidity prior to surgery, perhaps because clinicians did not consider it with the same significance.

Confidential: For Review Only

Another important example identified by many parents was the unanticipated psychological side effects on their child, the siblings and themselves, which were invariably lacking from discussions prior to cardiac surgery.

The lack of warning, and a consequent feeling of being completely unprepared to deal with these morbidities once they arose, was a clear difficulty expressed by parents.

- **Timing of consent**

The timing of consent conversations was also highlighted as being suboptimal. Examples were given of consent being taken the night before surgery, when parents were feeling not only anxious, but also left with little time to reflect on or think through what had been said. Parents identified that this affected their ability to understand and recall what they were told about the risks of surgery, contributing to making a difficult time more challenging.

3.Information needs of families

- **There is no right amount of information**

It was clear that there was no 'right amount of information' that suits every individual. Whilst some parents wanted to know about all possibilities, others felt that too much information would have been overwhelming, potentially paralysing them in the decision-making process.

Participants felt that the detail of information being conveyed should be tailored according to the needs of the individual receiving it, rather than using a 'one size fits all' approach.

- **Types of information**

Confidential: For Review Only

Written and audio-visual material were reported as helpful supplements. Being able to take something back to their accommodation, that could be read and digested in their own time, was valued. As well as more high-tech information, simple leaflets were appreciated as something that could be read without the need for internet access and a phone/computer.

- **Access to staff / lay support**

Parents valued access to a member of staff they could contact with further questions, and who could signpost them to other information as needed.

In addition, parents expressed that being able to speak to another parent, who had gone through something similar, and who *actually knew* how it felt to be a parent going through this, would have been a source of comfort and help.

Discussion

Healthcare communication is a vital skill, influencing understanding of information and hence informed decision making. The importance of 'good communication' however goes beyond just understanding. Meert et al [8] ask whether the way in which clinicians communicate may also impact on the psychological adjustment and functioning of parents and families.

Our study describes clinician, parental (predominantly maternal) and situational factors that may influence parental understanding of potential morbidities following paediatric cardiac surgery, and represent areas that can be targeted to improve this (see Table 3):

Confidential: For Review Only

- Clinicians use of jargon, the lack of consistency between clinicians and an individual clinician's skill in communication have been previously identified as important factors in patient comprehension and adherence [26, 27] and in the current study all of these aspects impacted reported parental satisfaction and understanding of the conversation. Individual communication skills vary, with one participant noting that some clinicians are 'just better' at it than others. Though this may be an innate strength or weakness within an individual, clinicians need to reflect and improve upon their own communication skills, in the same way they would for any technical or procedural skills required of them. Observation of exemplar mentors and simulation training in communication including discussions with parents of children who have undergone cardiac surgery are useful strategies to improve these skills and gain parent centred perspectives. [28]

- We know that the amount of information given by clinicians can have both positive and negative effects on parental anxiety. [7, 29] This was also reflected in our findings, suggesting that information given to parents needs to be tailored to the information needs of the individual receiving it. Taking the time to know what is right for the parent a clinician is speaking to is an important investment not only in the parent's understanding, but also in their ability to make decisions and cope with their child's post-operative course. In addition, parents want to know what is likely to happen to *their* child, such that the risk of morbidities would ideally be tailored to the individual characteristics of the child for whom surgery is being discussed. However, as the population of cardiac children become increasingly complex, and as surgical and medical techniques evolve, tailoring assessments of risk is difficult and

https://mc.manuscriptcentral.com/bmjpo

Confidential: For Review Only

always subject to some uncertainty. How clinicians support parents and families in coping with uncertainty needs to be an important part of specialist care.

- Stress and time pressure are barriers to informed consent. [30, 31] Participants commented on the difficulties they faced with consent the night before surgery. Where possible, a staged consent approach [32] should be used, such that an initial consultation will be used just to relay information, followed by a period of time to allow consideration by the parents, before a second conversation where consent is actually sought.

Many of our participants commented on the feeling of being 'unprepared'. We suggest future work should seek to understand if clinicians are able to mitigate some of the short and long-term distress experienced by families in this situation, through better communication and understanding of the parental perspective of morbidities and their impact on the child and family.

Parents described the importance of other sources of information that they could access whenever they wanted to, such as written information and internet resources. Written information has been found to result in significant decreases in parental anxiety and improved parental comprehension and satisfaction prior to their child's surgery.[33] Multi-media based health information has also been found to reduce parental anxiety [34] although evidence supporting the greater effectiveness of any one method of information provision is lacking, [34, 35] highlighting the importance of individually tailoring information

https://mc.manuscriptcentral.com/bmjpo

Confidential: For Review Only

provision. Parents in our study also valued the support offered by staff and in particular the support of other parents who had been through similar experiences, replicating findings with other illness groups.[36]

In this study, the online forum gave us access to many more parents than the focus groups (72 online, 13 at focus groups). This correlates with other reports suggesting the relative ease of online discussion compared to attending in person focus groups. [11, 12, 21]

Limitations

The data we obtained relied on participants' retrospective recall of conversations (with its inherent limitations on accuracy). Future work to look at clinician – parent communication in real time, would provide a valuable insight into what is *actually* said, and the interpretation at the time - not only of parents, but also of clinicians – i.e. what parents understood from the conversation and what clinicians believe the parents understood from the conversation. Furthermore, using an online forum to collect parent perceptions does not result in the same depth of information that would be possible in individual interviews and the approach precludes probing for further detail.

We recognise that both the online forum and the focus groups can in themselves exclude certain individuals due to lack of familiarity, language barriers, literacy barriers and lack of internet resources. In addition, our study lacked the views of males and non-Caucasians, who were under-represented in our participant sample, despite the chosen locations for the focus groups having a broad ethnic and socio-economic mix. The challenges of including

Confidential: For Review Only

ethnically, culturally and linguistically diverse populations, as well as fathers, in paediatric research studies are well documented. [37, 38] In order to capture these important often unheard views, we must find and adopt an innovative approach that successfully includes minority groups to ensure the broadest capture of parental/family views. Despite specifically choosing a data collection method to increase the accessibility of the research to potential participants, the fact that our participants did not reflect a broad range of ethnic groups or gender also limits the transferability of our findings to the wider population of parents of children with congenital heart disease in the UK.

Finally, we did not collect the same demographic information from participants in the online forum and the focus groups, which limits our ability to describe certain aspects of our participants across both data collection approaches.

Conclusions and suggestions for future practice

We aimed to elicit parents' perceptions about health professionals' communication regarding morbidities associated with paediatric cardiac surgery. The nature of the online forum, a relatively novel approach to collecting data from parents in a research context, and to a lesser extent the focus groups, promoted free debate and sharing of views with a large group of parents/carers, providing data that may not have been obtained in interviews.

The feedback we obtained suggests that there is wide variability and lack of consistency in the way clinicians describe complications, much of which may not be absorbed or retained by parents, particularly during times of extreme stress and distress. [28, 39, 40] A recent

https://mc.manuscriptcentral.com/bmjpo

Confidential: For Review Only

survey exploring communication between parents and clinical teams following children's heart surgery [40] supports our findings with reports of inconsistent communication (almost two-thirds of parents reporting this), particularly where complications arose or children were in hospital for longer periods of time.

Analysis of the data points towards the need for an individualised approach to communication around complications, based on 'high quality conversations', in which certain *key principles* of good communication are followed, as discussed above. However, it is also important to recognise that this is not something which is unique to paediatric cardiac surgery – rather it is relevant in any situation in which an individual is undergoing a medical or surgical intervention.

Future work to investigate the impact of 'good communication' on the short- and long-term psychological morbidity of parents and families would be a valuable next step, together with identifying how this may impact on physical and psychological outcomes in the children undergoing surgery.

Confidential: For Review Only

Funding details

This project was funded by the National Institute for Health Research Health Services and Delivery Research programme (Project No: 12/5005/06).

Acknowledgement

We would like to thank the Children's Heart Federation and Ms. Rehana Ahmed for their assistance in moderating the online forum and focus groups.

Research at Great Ormond Street Hospital is supported by the Great Ormond Street Hospital National Institute of Health Research Biomedical Research Centre.

Contributor statement

K Brown, C Pagel and J Wray planned the study. The forum and focus groups were conducted by R Ahmed and the Children's Heart Federation. V Rajagopal, C Pagel and J Wray analysed and reported results. V Rajagopal wrote the first draft of the manuscript and all authors approved the final version.

Confidential: For Review Only

What is already known about this topic:

- Stress, time pressure and 'quality' of communication can adversely affect parental understanding, potentially limiting their ability to make informed decisions.

What this study adds:

- We have identified a range of clinician, parental and situational factors that may influence parental understanding during communication about morbidity following paediatric cardiac surgery.
- These factors represent targets for improvement and have led to the development of our principles of 'good communication', described in this paper.

Confidential: For Review Only

References

1. Brown KL, Crowe S, Franklin R, McLean A, Cunningham D, Barron D, et al. Trends in 30-day mortality rate and case mix for paediatric cardiac surgery in the UK between 2000 and 2010. Open Heart. 2015;2(1):e000157. Epub 2015/04/22. doi: 10.1136/openhrt-2014-000157. PubMed PMID: 25893099; PubMed Central PMCID: PMCPMC4395835.
2. National Congential Heart Disease Annual Report 2013-2016. Available from: https://www.ucl.ac.uk/nicor/audits/congenital/documents/annual-reports/National_Congenital_Heart_Disease_2013_16_annual_report.
3. NIHR Evaluation TaS. [11 June 2018]. Available from: http://www.nets.nihr.ac.uk/projects/hsdr/12500506.
4. Brown KL, Ridout D, Pagel C, Wray J, Anderson D, Barron DJ, et al. Incidence and risk factors for important early morbidities associated with pediatric cardiac surgery in a UK population. J Thorac Cardiovasc Surg. 2019;158(4):1185-96 e7. Epub 2019/07/30. doi: 10.1016/j.jtcvs.2019.03.139. PubMed PMID: 31353100.
5. Levi RB, Marsick R, Drotar D, Kodish ED. Diagnosis, disclosure, and informed consent: learning from parents of children with cancer. J Pediatr Hematol Oncol. 2000;22(1):3-12. Epub 2000/03/01. doi: 10.1097/00043426-200001000-00002. PubMed PMID: 10695815.
6. Sloper P. Predictors of distress in parents of children with cancer: a prospective study. J Pediatr Psychol. 2000;25(2):79-91. Epub 2000/05/23. doi: 10.1093/jpepsy/25.2.79. PubMed PMID: 10820946.
7. Miller VA, Drotar D, Burant C, Kodish E. Clinician-parent communication during informed consent for pediatric leukemia trials. J Pediatr Psychol. 2005;30(3):219-29. Epub 2005/03/24. doi: 10.1093/jpepsy/jsi032. PubMed PMID: 15784918.
8. Meert KL, Eggly S, Pollack M, Anand KJ, Zimmerman J, Carcillo J, et al. Parents' perspectives on physician-parent communication near the time of a child's death in the pediatric intensive care unit. Pediatr Crit Care Med. 2008;9(1):2-7. Epub 2008/05/15. doi: 10.1097/01.PCC.0000298644.13882.88. PubMed PMID: 18477906; PubMed Central PMCID: PMCPMC3198033.
9. Pagel C, Brown KL, McLeod I, Jepps H, Wray J, Chigaru L, et al. Selection by a panel of clinicians and family representatives of important early morbidities associated with paediatric cardiac surgery suitable for routine monitoring using the nominal group technique and a robust voting process. BMJ Open. 2017;7(5):e014743. Epub 2017/05/31. doi: 10.1136/bmjopen-2016-014743. PubMed PMID: 28554921; PubMed Central PMCID: PMCPMC5729972.
10. Brown KL, Pagel C, Brimmell R, Bull K, Davis P, Franklin RC, et al. Definition of important early morbidities related to paediatric cardiac surgery. Cardiol Young. 2017;27(4):747-56. Epub 2016/09/30. doi: 10.1017/S1047951116001256. PubMed PMID: 27680207.
11. De Simoni A, Shanks A, Mant J, Skelton JR. Making sense of patients' internet forums: a systematic method using discourse analysis. Br J Gen Pract. 2014;64(620):e178-80. Epub 2014/02/26. doi: 10.3399/bjgp14X677671. PubMed PMID: 24567657; PubMed Central PMCID: PMCPMC3933853.
12. Balasooriya-Smeekens C, Bateman A, Mant J, De Simoni A. Barriers and facilitators to staying in work after stroke: insight from an online forum. BMJ Open. 2016;6(4):e009974. Epub 2016/04/08. doi: 10.1136/bmjopen-2015-009974. PubMed PMID: 27053267; PubMed Central PMCID: PMCPMC4823433.
13. Im EO, Chee W. An online forum as a qualitative research method: practical issues. Nurs Res. 2006;55(4):267-73. Epub 2006/07/20. PubMed PMID: 16849979; PubMed Central PMCID: PMCPMC2491331.
14. Im EO, Chee W. Practical guidelines for qualitative research using online forums. Comput Inform Nurs. 2012;30(11):604-11. Epub 2012/08/25. doi: 10.1097/NXN.0b013e318266cade. PubMed PMID: 22918135; PubMed Central PMCID: PMCPMC3727223.

Confidential: For Review Only

15. Alinia H, Moradi Tuchayi S, Farhangian ME, Huang KE, Taylor SL, Kuo S, et al. Rosacea patients seeking advice: Qualitative analysis of patients' posts on a rosacea support forum. J Dermatolog Treat. 2016;27(2):99-102. Epub 2016/01/28. doi: 10.3109/09546634.2015.1133881. PubMed PMID: 26815357.
16. Jamison J, Sutton S, Mant J, De Simoni A. Online stroke forum as source of data for qualitative research: insights from a comparison with patients' interviews. BMJ Open. 2018;8(3):e020133. Epub 2018/04/01. doi: 10.1136/bmjopen-2017-020133. PubMed PMID: 29602848; PubMed Central PMCID: PMCPMC5884329.
17. Kaufman S, Whitehead KA. Producing, ratifying, and resisting support in an online support forum. Health (London). 2018;22(3):223-39. Epub 2016/02/07. doi: 10.1177/1363459315628043. PubMed PMID: 26851264.
18. Malik S, Coulson NS. The therapeutic potential of the internet: exploring self-help processes in an internet forum for young people with inflammatory bowel disease. Gastroenterol Nurs. 2011;34(6):439-48. Epub 2011/12/02. doi: 10.1097/SGA.0b013e318237a9ba. PubMed PMID: 22129797.
19. McKechnie V, Barker C, Stott J. The effectiveness of an Internet support forum for carers of people with dementia: a pre-post cohort study. J Med Internet Res. 2014;16(2):e68. Epub 2014/03/04. doi: 10.2196/jmir.3166. PubMed PMID: 24583789; PubMed Central PMCID: PMCPMC3961748.
20. Zhao J, Abrahamson K, Anderson JG, Ha S, Widdows R. Trust, empathy, social identity, and contribution of knowledge within patient online communities. Behaviour & Information Technology. 2013;32:1041-8. doi: 10.1080/0144929X.2013.819529.
21. Rhodes SD, Bowie DA, Hergenrather KC. Collecting behavioural data using the world wide web: considerations for researchers. J Epidemiol Community Health. 2003;57(1):68-73. Epub 2002/12/20. PubMed PMID: 12490652; PubMed Central PMCID: PMCPMC1732282.
22. Wray J, Brown K, Tregay J, Crowe S, Knowles R, Bull K, et al. Parents' Experiences of Caring for Their Child at the Time of Discharge After Cardiac Surgery and During the Postdischarge Period: Qualitative Study Using an Online Forum. J Med Internet Res. 2018;20(5):e155. Epub 2018/05/11. doi: 10.2196/jmir.9104. PubMed PMID: 29743157; PubMed Central PMCID: PMCPMC5966654.
23. . Available from: http://www.chfed.org.uk/parents-wanted-for-a-study-looking-into-complications-following-heart-surgery/.
24. Coffey A, Atkinson P. Making Sense of Qualitative Data:Complementary Research Strategies. Thousand Oaks, CA: Sage Publications; 1996.
25. Braun V, Clarke V. Using thematic analysis in psychology. Qualitative Research in Psychology. 2006;3:77-101.
26. Castro CM, Wilson C, Wang F, Schillinger D. Babel babble: physicians' use of unclarified medical jargon with patients. Am J Health Behav. 2007;31 Suppl 1:S85-95. Epub 2007/10/20. doi: 10.5555/ajhb.2007.31.supp.S85. PubMed PMID: 17931142.
27. Zolnierek KB, Dimatteo MR. Physician communication and patient adherence to treatment: a meta-analysis. Med Care. 2009;47(8):826-34. Epub 2009/07/09. doi: 10.1097/MLR.0b013e31819a5acc. PubMed PMID: 19584762; PubMed Central PMCID: PMCPMC2728700.
28. Levetown M, American Academy of Pediatrics Committee on B. Communicating with children and families: from everyday interactions to skill in conveying distressing information. Pediatrics. 2008;121(5):e1441-60. Epub 2008/05/03. doi: 10.1542/peds.2008-0565. PubMed PMID: 18450887.
29. Pomicino L, Maccacari E, Buchini S. Levels of anxiety in parents in the 24 hr before and after their child's surgery: A descriptive study. J Clin Nurs. 2018;27(1-2):278-87. Epub 2017/05/26. doi: 10.1111/jocn.13895. PubMed PMID: 28544343.
30. Barnlund DC. The mystification of meaning: doctor-patient encounters. J Med Educ. 1976;51(09):716-25. Epub 1976/09/01. PubMed PMID: 957387.

Confidential: For Review Only

31. Janis I. Decision making under stress. In: Goldberger L, Breznitz S, editors. Handbook of stress : theoretical and clinical aspects. New York: The Free Press; 1993. p. 56-74.
32. Angiolillo AL, Simon C, Kodish E, Lange B, Noll RB, Ruccione K, et al. Staged informed consent for a randomized clinical trial in childhood leukemia: impact on the consent process. Pediatr Blood Cancer. 2004;42(5):433-7. Epub 2004/03/30. doi: 10.1002/pbc.20010. PubMed PMID: 15049015.
33. Landier M, Villemagne T, Le Touze A, Braik K, Meignan P, Cook AR, et al. The position of a written document in preoperative information for pediatric surgery: A randomized controlled trial on parental anxiety, knowledge, and satisfaction. J Pediatr Surg. 2018;53(3):375-80. Epub 2017/05/01. doi: 10.1016/j.jpedsurg.2017.04.009. PubMed PMID: 28456425.
34. Botngard A, Skranes LP, Skranes J, Dollner H. Multimedia based health information to parents in a pediatric acute ward: a randomized controlled trial. Patient Educ Couns. 2013;93(3):389-93. Epub 2013/05/21. doi: 10.1016/j.pec.2013.04.017. PubMed PMID: 23684375.
35. Franck LS, Spencer C. Informing parents about anaesthesia for children's surgery: a critical literature review. Patient Educ Couns. 2005;59(2):117-25. Epub 2005/11/01. doi: 10.1016/j.pec.2004.11.002. PubMed PMID: 16257615.
36. Shilling V, Morris C, Thompson-Coon J, Ukoumunne O, Rogers M, Logan S. Peer support for parents of children with chronic disabling conditions: a systematic review of quantitative and qualitative studies. Dev Med Child Neurol. 2013;55(7):602-9. Epub 2013/02/21. doi: 10.1111/dmcn.12091. PubMed PMID: 23421818.
37. Costigan CL, Cox MJ. Fathers' participation in family research: is there a self-selection bias? J Fam Psychol. 2001;15(4):706-20. Epub 2002/01/05. PubMed PMID: 11770476.
38. Williams A, Oulton K, Sell D, Wray J. Healthcare professional and interpreter perspectives on working with and caring for non-English speaking families in a tertiary paediatric healthcare setting. Ethn Health. 2017:1-14. Epub 2017/03/10. doi: 10.1080/13557858.2017.1294662. PubMed PMID: 28277020.
39. Scrimin S, Haynes M, Altoe G, Bornstein MH, Axia G. Anxiety and stress in mothers and fathers in the 24 h after their child's surgery. Child Care Health Dev. 2009;35(2):227-33. Epub 2009/02/21. doi: 10.1111/j.1365-2214.2008.00920.x. PubMed PMID: 19228156; PubMed Central PMCID: PMCPMC3496245.
40. Pagel C, Bull C, Utley M, Wray J, Barron DJ, Stoica S, et al. Exploring communication between parents and clinical teams following children's heart surgery: a survey in the UK. BMJ Paediatr Open. 2019;3(1):e000391. Epub 2019/06/18. doi: 10.1136/bmjpo-2018-000391. PubMed PMID: 31206072; PubMed Central PMCID: PMCPMC6542420.

Confidential: For Review Only

Table 1: Questions asked on the online forum

**1. When thinking about children's heart surgery, what does the word "complication" mean to you?**
**2. When thinking about children's heart surgery, what does the word 'morbidity' mean to you? Can you give an example of how this affected you or your child?**
**3. As the experience of heart surgery becomes more distant and in the past, and your child is older, are there any difficulties that your child has now which you think may relate to the operation?**
**4. Thinking about yourself and your family, can you help us understand the impact that any complications had on you, your child or any other family members:**<br>➢ **While your child was still in hospital?**<br>➢ **After you got home?**
**6. We are thinking about measuring and recording how often complications happen, but we want to concentrate on the things that matter most to the child and family. Here is a list of problems that we know can happen:**<br>• *Children who have heart problems may sometimes experience different types of brain damage and this may lead to disability ranging from loss of hearing – to problems with learning or movement or all types of disability.*<br>• *A child may sometimes need an extra operation during the same stay in hospital that was not planned at the start.*<br>• *Infection may sometimes happen after heart surgery.*<br>• *A child's kidneys may stop working and need to be supported with a machine for a period of time.*<br>• *The muscle that helps a child to breathe may be weakened because the nerve supplying it is bruised or damaged – sometimes an operation is needed to strengthen the breathing muscle (diaphragm muscle).*<br>• *A child may need to stay in intensive care for a long time because he / she needed assistance with breathing or he / she needed tubes to remain in place.*<br>• *A child's gut may not digest milk or food and he / she may need to have nutrition via a drip.*<br>• *A child's heart may become very weak such that a machine to support the body called 'ECMO' was needed.*<br>• *Damage can happen to the nerve supply of the heart so that the child needs to have a pace maker put in to regulate the heart-beat.*<br>➢ **Which of these complications seems the worst or most worrying to you?**

Confidential: For Review Only

**7. We are thinking about measuring and recording how often complications happen, but we want to concentrate on the things that matter most to the child and family. Here is a list of problems that we know can happen:**<br>• *Children who have heart problems may sometimes experience different types of brain damage and this may lead to disability ranging from loss of hearing – to problems with learning or movement or all types of disability.*<br>• *A child may sometimes need an extra operation during the same stay in hospital that was not planned at the start.*<br>• *Infection may sometimes happen after heart surgery.*<br>• *A child's kidneys may stop working and need to be supported with a machine for a period of time.*<br>• *The muscle that helps a child to breathe may be weakened because the nerve supplying it is bruised or damaged – sometimes an operation is needed to strengthen the breathing muscle (diaphragm muscle).*<br>• *A child may need to stay in intensive care for a long time because he / she needed assistance with breathing or he / she needed tubes to remain in place.*<br>• *A child's gut may not digest milk or food and he / she may need to have nutrition via a drip.*<br>• *A child's heart may become very weak such that a machine to support the body called 'ECMO' was needed.*<br>• *Damage can happen to the nerve supply of the heart so that the child needs to have a pace maker put in to regulate the heart-beat.*<br>➢ **Could you please tell us why you think one or more of these complications is 'worse' than other complications?**<br>➢ **Are there things that happened to your child after their operation that you think 'went wrong' which should be on this list but isn't?**
**8. Could you please let us know what information you were provided with regarding the complications of your child's heart surgery?**
**9. (a) Do you feel you were provided with the right amount of information regarding complications before surgery?**<br>**9. (b) How much information would you like (more or less)? How much detail is helpful?**
**10. What visual (books etc) methods could be developed for letting families know the risk of complications to better inform them about what might happen after surgery?**

Confidential: For Review Only

**11. What do you think about everyone being able to see (on the internet) the numbers of complications occurring after children's heart surgery at different hospitals?**
**12. (a) Do you have any further comments regarding complications following heart surgery? Is there anything we should know to improve services? Is there anything else you can think of?**<br>**12. (b) Is there anything we could have done to improve the online study? Do you think we should have asked questions more frequently, for example? Any other suggestions?**

Confidential: For Review Only

Table 2: Themes and sub-themes

Themes	Sub-themes	Quotes
**1.Clinicians' use of language**	***Comprehension***	▪ *"It's really easy to get yourself tied up in knots with risks and percentages, when they're really not explained properly"*<br>▪ *"Percentages are rarely accurate anyway, so why do we - as parents - hang on to them so dearly (me included!!!!). We were given 13% but it wasn't properly explained that this was risks (i.e. morbidity) not death alone."*<br>▪ *"I think it's assumed that parents have a basic understanding of statistics when it's such a complicated measurement and it's not properly explained to us."*<br>▪ *"It's just jargon...Please...English...so we can understand it!"*<br>▪ *"…some doctors are better than others...at explaining"*<br>▪ *"they go into the usual doctor terminology" "they use hypoplastic instead of small, stenotic instead of stiff"*<br>▪ *"Morbidity to me means death."*<br>▪ *'Just to talk mum to mum…then you're not getting the medical jargon'*
	Consistency	▪ *'I found a huge discrepancy between the way the cardiologists describe surgery i.e. very optimistic, complications are rare, and the surgeons who spell it out in order to cover themselves. Personally, I prefer the latter as it means when it does happen you are aware of it and know it has happened before, whereas the former makes you feel so unlucky and wondering why things have happened'.*<br>▪ *"We were given very detailed information by the surgeons on the eve of our sons' op but ... up until that time we had only seen cardiologists who were really quite blasé…We were told 'he'll be fine' they do switches all the time. Turns out it was far from that..."*
**2.Being unprepared for complications**	***Differing priorities of Health Care Professionals (HCP) and families***	▪ *"NG feeding was never something I thought about when we considered the prospect of having a congenital heart disease child."*<br>▪ *"We weren't mentally prepared for the longer stay as we were told 'in and out in 5 days'."*<br>▪ *"I really wish someone had prepared me for the psychological side effects... anything explaining how trauma and complications can have a negative impact on your child's self-esteem and mental well-being"*<br>▪ *"Tell parents beforehand…This is very likely going to affect development in growth, height, learning and development and things like that"*<br>▪ *"They do tell you some of the physical things that might happen" but not "how it might affect…a person's behaviour or emotions"*

Confidential: For Review Only

	Timing of consent	▪ *"You know when they do the consent forms it's usually the night before surgery which...is a bad idea, because you're not taking that in... if you did it a week before...you take in a lot more... It's easy for you to digest and understand"*
**3.Information needs of families**	***No right amount of information***	▪ *"Some parents will want to know everything and others want to know as little as possible"*<br>▪ *"If we knew all the potential outcomes I think signing the consent would have been so much harder"*<br>▪ *"You can't have a blanket rule of 'We must tell them every possible thing that could go wrong' or, 'We only tell them the most common'. You need to look at it case-by-case"*<br>▪ *"It's such a delicate balance…you've got to try and test the waters with the patient's parents and the patient's themselves to find out how much information...you need to give this person. It has to be an individual case-by-case scenario"*
	Types of information	▪ *"Something to take away and look at and digest in your own time"*<br>▪ *"Even a simple information sheet…you might not have internet access…you might need to go into your room or sit by the bed and have another look at it a bit later"*<br>▪ *"Little Hearts Matter...DVD pack for antenatal diagnosis. It was brilliant."*
	**Access to staff / lay support**	▪ *"Community Liaison Nurses are very useful and parents should be given contact numbers as a matter of course "*<br>▪ *"knowing who to ask…that there's somebody you can ask about things you might spot…signposting"*

Confidential: For Review Only

Table 3: Principles of good communication

1.	Openness
2.	Avoid jargon
3.	Include emotional and developmental complications during consent
4.	Consistent communication within and across all teams
5.	Appropriate timing of conversations
6.	Tailor conversations to the individual in front of you
7.	Individualise risk – what is likely for *this* particular child?
8.	Provide written and audio-visual material
9.	Provide a liaison link professional with contacts to parental support groups

Confidential: For Review Only

Confidential: For Review Only

Figure 1: Summary of feedback from parents about how complications were communicated to them by healthcare professionals

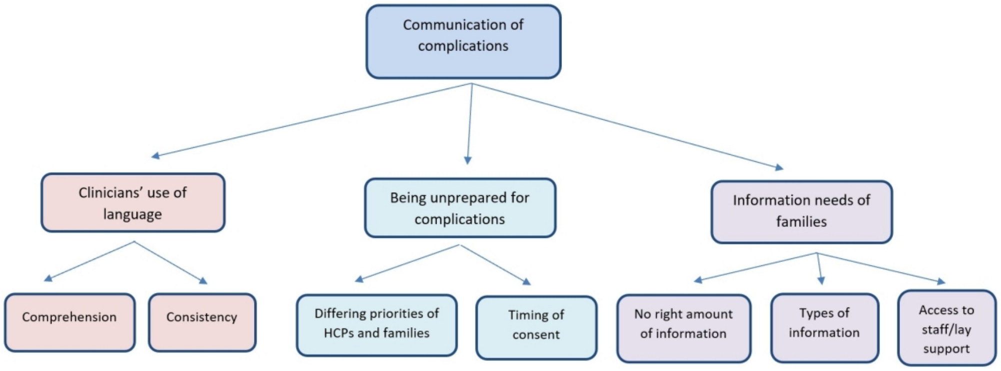

527x240mm (96 x 96 DPI)

BMJ Paediatrics Open

Parental understanding of our communication of morbidity associated with paediatric cardiac surgery: a qualitative study.

Journal:	*BMJ Paediatrics Open*
Manuscript ID	bmjpo-2019-000578.R2
Article Type:	Original article
Date Submitted by the Author:	11-Jan-2020
Complete List of Authors:	Rajagopal, Veena; Great Ormond Street Hospital For Children NHS Foundation Trust,<br>Brown, Katherine; Great Ormond Street Hospital For Children NHS Foundation Trust<br>Pagel, Christina; University College London; Great Ormond Street Hospital For Children NHS Foundation Trust<br>Wray, Jo; Great Ormond Street Hospital For Children NHS Foundation Trust
Keywords:	Cardiac Surgery, Intensive Care, Qualitative research, Outcomes research, Psychology

SCHOLARONE™
Manuscripts

I, the Submitting Author has the right to grant and does grant on behalf of all authors of the Work (as defined in the below author licence), an exclusive licence and/or a non-exclusive licence for contributions from authors who are: i) UK Crown employees; ii) where BMJ has agreed a CC-BY licence shall apply, and/or iii) in accordance with the terms applicable for US Federal Government officers or employees acting as part of their official duties; on a worldwide, perpetual, irrevocable, royalty-free basis to BMJ Publishing Group Ltd ("BMJ") its licensees and where the relevant Journal is co-owned by BMJ to the co-owners of the Journal, to publish the Work in this journal and any other BMJ products and to exploit all rights, as set out in our licence.

The Submitting Author accepts and understands that any supply made under these terms is made by BMJ to the Submitting Author unless you are acting as an employee on behalf of your employer or a postgraduate student of an affiliated institution which is paying any applicable article publishing charge ("APC") for Open Access articles. Where the Submitting Author wishes to make the Work available on an Open Access basis (and intends to pay the relevant APC), the terms of reuse of such Open Access shall be governed by a Creative Commons licence – details of these licences and which Creative Commons licence will apply to this Work are set out in our licence referred to above.

Other than as permitted in any relevant BMJ Author's Self Archiving Policies, I confirm this Work has not been accepted for publication elsewhere, is not being considered for publication elsewhere and does not duplicate material already published. I confirm all authors consent to publication of this Work and authorise the granting of this licence.

Confidential: For Review Only

Parental understanding of our communication of morbidity associated with paediatric cardiac surgery: a qualitative study

Veena Rajagopal [1], Katherine Brown [1], Christina Pagel [2], Jo Wray [1]

[1] Heart and Lung, Great Ormond Street Hospital for Children NHS Foundation Trust, London, United Kingdom

[2] Clinical Operational Research Unit, University College London, United Kingdom

Address for correspondence
Dr Jo Wray
Critical Care and Cardiorespiratory Directorate
Great Ormond Street Hospital
Great Ormond Street
London WC1N 3JH

Email: jo.wray@gosh.nhs.uk

Key words: parents, cardiac surgery, morbidity, communication

Confidential: For Review Only

Abstract

Background: Following paediatric cardiac surgery, quality of life may be significantly impacted by morbidities associated with cardiac surgery. Parental understanding of the potential for postoperative morbidity is important for informed decision making. As part of a broader research study, we aimed to elicit parental understanding and experience of the communication of morbidities following their child's cardiac surgery, using traditional focus groups together with an online forum.

Methods: The Children's Heart Federation set up and moderated a closed, anonymous online discussion group via their Facebook page, focusing on complications, information needs and methods of providing families with information. Additionally, we ran three focus groups with parents/carers, moderated by an experienced independent professional. Focus groups were recorded and transcribed and a single transcript was generated from the online forum. All transcripts were thematically analysed.

Results: All data were collected in 2014. The forum ran over 3 months in 2014 and involved 72 participants. Focus groups involved 13 participants. Three broad themes were identified: 1) Clinicians' use of language 2) Feeling unprepared for complications and 3) Information needs of families.

Conclusions: Clinicians' language is often misunderstood, with wide variability in the way morbidities are described, and between differing teams looking after the same child. Information may not be easily absorbed or retained by families, who often felt unprepared for morbidities that arose after their child's heart surgery. Here we propose key principles of good communication tailored to the individual receiving it.

Confidential: For Review Only

Introduction

A large majority of children now survive into adulthood following paediatric cardiac surgery, [1, 2] making it increasingly important to consider any associated morbidities (as described previously [3]). Furthermore, greater numbers of children with increasingly complex heart disease are now undergoing surgery, resulting in a growing number of children and families living with the impact of a range of morbidities arising as a consequence.[4] For those with the most severe morbidities the impact may be life changing or, in some cases, life-limiting. It is therefore imperative that information about morbidities is communicated in a clear and empathic way.

Very little is known about parental experience and understanding of our communication of morbidity associated with paediatric cardiac surgery. We do know from work in other settings however [5, 6], that levels of distress, time pressure and the quality of 'clinician-parent communication' can adversely affect parental understanding of information given, with the potential to limit parents' ability to make informed decisions. [7] Parents want their clinicians to be accessible, honest, caring, and use lay language at a pace that can be understood. [8]

As part of a wider study looking at the selection, definition, incidence and impact of morbidities following paediatric cardiac surgery [3, 4, 9, 10] our objective was to elicit parents' perceptions about the way healthcare professionals communicate regarding morbidities associated with paediatric cardiac surgery.

Traditional methods of accessing parental views may exclude certain groups including those who are more geographically remote and those from culturally and ethnically diverse

Confidential: For Review Only

backgrounds. [11, 12] Logistics, time, and discomfort in face-to-face and group interaction may deter some individuals. Online forums are an important way in which internet users seek health care information and communicate with others who have similar health care experiences. There is an increasing focus on understanding the potential value of this method of data collection in qualitative research. [13, 14] Forums have successfully been used in healthcare research, [15-20] providing a flexible and safe space, where open and honest discussions can be held over a period of months, in a conveniently non-synchronous way for potentially large numbers of users in different geographical areas. [11, 12, 21] There is no burden on travel, and participation results in minimal disruption to daily life.

By including an online forum as a method of engaging with families, alongside more traditional approaches, we hoped to access the views of those who find it hard to attend in person or take part in interviews and focus groups, adopting a similar approach to that used by our group previously. [22] We report here views elicited from a charity-moderated online forum and from three focus groups.

Confidential: For Review Only

Methods

Design

A qualitative approach underpinned by an interpretivist framework, employing an online forum in addition to three more traditional focus groups was used as a method of data collection, to access participant viewpoints.

Participants and data collection

The Children's Heart Federation (CHF), a national parent charity, facilitated and moderated a closed, anonymous, online discussion group via their Facebook page, following a similar approach to that reported in a previous separate study.[22] The discussion group was advertised on the charity's home web page [23] and anyone who had experienced taking care of a child after heart surgery was eligible to participate. Potential participants were directed to the charity's Facebook page where they could access information about the study and governance surrounding it. Anyone wishing to participate was asked to provide basic demographic details (age, gender, ethnicity and geographical region) and on completion of this they were directed to the closed Facebook group, where they were able to respond to questions posted there. The research team provided questions to be posted on the forum at the start of the process and the charity were responsible for deciding when new questions should be posted or any prompts introduced, based on participant responses and the rate of responding. New questions were introduced when no new information was being posted – i.e. when data saturation had been reached for each question. Questions posted on the forum are shown in Table 1. Questions were developed by the research team to address the overall aim of understanding parents' perceptions of how clinicians communicate about morbidities, which morbidities parents think are most important and should be measured and what information they had been given about morbidities prior to their child's surgery. The development of the questions was an iterative process and involved patient representatives who were members of the study team as well as health professionals in order to ensure that content and wording of questions were appropriate. The forum took place over a three-month period in 2014.

https://mc.manuscriptcentral.com/bmjpo

Confidential: For Review Only

In addition, we held three focus groups, which took place in Glasgow, Birmingham, and London. This additional method of participation was included to enable those who wanted to participate via a more traditional face-to-face approach to do so. These locations were chosen for their broad case mix and ethnic diversity. The focus groups were advertised via the CHF website (as for the online forum) [23] and potential participants contacted the CHF if they wanted to take part. The CHF organised the groups, which were held on a Saturday and were moderated by an independent, experienced researcher using as a framework the same questions that were used for the online forum (see table 1). Focus group participants provided written consent for their participation, recording and use of anonymised quotes in dissemination of the findings. Each focus group was audio-recorded and transcribed verbatim.

Ethical approval was granted by London City Road Research Ethics Committee (study number: 14-LO-1442).

Data management and analysis

Responses from the online forum were collated into a single transcript. The transcripts from the online forum and focus groups were thematically analysed, [24] enabling identification, analysis and reporting of patterns within the data [25] related to parental understanding and experience of the communication of morbidities following their child's cardiac surgery. Non-parent/carer responses were excluded from analysis. Transcripts were read and codes attached to segments of data independently by members of the research team (JW/CP/VR). Similar codes were then merged to create themes. The researchers met to discuss the themes and

Confidential: For Review Only

agree the descriptive names assigned to them. Discussion continued until consensus was reached.

Participants in the online forum were unknown and not identifiable by the research team, therefore there was no involvement of participants in transcript review and coding.

Patient and Public Involvement

In the broader study, the list of morbidities linked to paediatric cardiac surgery was prioritised by a panel reflecting the views of professionals, parents and patients. This list was used in the questions posted on the forum and at the focus groups.

The reporting of these data is motivated by families reporting inconsistencies in the communication of morbidity related to cardiac surgery.

Results

Demographics of participants

- ***Online forum***

The forum ran over 3 months in 2014 and involved 72 participants (68 mothers, 1 father, 1 patient, 2 grandmothers; age 15-59 years). The vast majority of participants were white British (n=70; 97%) but there was a spread of participants across England, Wales and Scotland.

- ***Focus groups***

The three focus groups took place in 2014, each lasting approximately two hours and in total comprised 13 participants (10 mothers, 2 fathers, and 1 adult congenital heart disease patient).

Confidential: For Review Only

Everyone who expressed an interest in attending a focus group was able to do so, although some were not able to attend on the day due to other commitments or their child being unwell. The ages of the parents' children ranged from 14 months - 24 years (however only one 'child' was an adult, the others all being 14 years old or younger, with a median age of 5 years at the time of the group). The gender of the participants' children was 7 boys and 5 girls. Two children had recognised syndromes, and one of the mothers was bereaved.

Collated online forum and focus group data

A number of codes were identified from the online forum and focus group data, and collated to form three themes, with seven subthemes, relating to communication between healthcare professionals and families about complications arising from cardiac surgery. The themes are shown in Tables 2-4, with illustrative quotes, and explained in more detail below.

Themes

Figure 1 illustrates the three themes with their related subthemes.

1.Clinicians' use of language

- **Comprehension**

Language used by clinicians about complications of surgery was often poorly understood by families. Jargon (e.g. words such as 'morbidity', 'stenotic', 'hypoplastic') was used instead of lay language, and percentages were used to communicate risk. These were often confusing to participants and led to misunderstanding.

Confidential: For Review Only

Some parents felt it would have been useful to hear from another parent who had been through a similar experience before, someone who could tell them 'mum to mum' with 'no jargon', what to expect.

- **Consistency**

Participants described different specialties as giving different messages about which morbidities might happen following surgery. Whilst some played down the chance of morbidities happening, others were seen as being more up-front during consent conversations. These inconsistencies between teams led to mistrust, and a perception that some clinicians were being more honest than others.

Confidential: For Review Only

2.Being unprepared for complications

- **Differing priorities of health care professionals and families**

Parents often felt unprepared for morbidities when they arose. Differing priorities between clinical teams and families may have resulted in some morbidities not being discussed prior to surgery. For example, feeding difficulties, and the need for a nasogastric tube were of huge significance to families, however parents were frequently not made aware of this as a potential morbidity, perhaps because clinicians did not consider it with the same significance.

Another important example identified by many parents was the unanticipated psychological side effects on their child, the siblings and themselves, which were invariably lacking from discussions prior to cardiac surgery.

The lack of warning, and a consequent feeling of being completely unprepared to deal with these morbidities once they arose, was a clear difficulty expressed by parents.

- **Timing of consent**

The timing of consent conversations was also highlighted as being suboptimal. Examples were given of consent being taken the night before surgery, when parents were feeling not only anxious, but also left with little time to reflect on or think through what had been said. Parents identified that this affected their ability to understand and recall what they were told about the risks of surgery, contributing to making a difficult time more challenging.

3.Information needs of families

- **There is no right amount of information**

It was clear that there was no 'right amount of information' that suits every individual. Whilst some parents wanted to know about all possibilities, others felt that too much information would have been overwhelming, potentially paralysing them in the decision-making process.

Participants felt that the detail of information being conveyed should be tailored according to the needs of the individual receiving it, rather than using a 'one size fits all' approach.

- **Types of information**

Written and audio-visual material were reported as helpful supplements. Being able to take something back to their accommodation, that could be read and digested in their own time, was valued. As well as more high-tech information, simple leaflets were appreciated as something that could be read without the need for internet access and a phone/computer.

Confidential: For Review Only

- **Access to staff / lay support**

Parents valued access to a member of staff they could contact with further questions, and who could signpost them to other information as needed.

In addition, parents expressed that being able to speak to another parent, who had gone through something similar, and who *actually knew* how it felt to be a parent going through this, would have been a source of comfort and help.

Discussion

Healthcare communication is a vital skill, influencing understanding of information and hence informed decision making. The importance of 'good communication' however goes beyond just understanding and it has been suggested that the way in which clinicians communicate may also impact on the psychological adjustment and functioning of parents and families. [8] It is widely acknowledged that paediatric cardiac surgery is very stressful for parents, particularly in view of the risk of morbidities associated with the operation, and how clinicians communicate is likely to have an impact on parents. We therefore wanted to explore parents' perceptions of how clinicians communicate about morbidities and what information they had been given about morbidities prior to their child's surgery. The feedback we obtained suggests that there is wide variability and lack of consistency in the way clinicians describe morbidities, much of which may not be absorbed or retained by parents, particularly during times of extreme stress and distress. [26, 27, 28] A recent survey exploring communication between parents and clinical teams following children's heart surgery [28] supports our findings with reports of inconsistent

Confidential: For Review Only

communication (almost two-thirds of parents reporting this), particularly where complications arose or children were in hospital for longer periods of time.

Our study describes clinician, parental (predominantly maternal) and situational factors that may influence parental understanding of potential morbidities following paediatric cardiac surgery, representing areas that can be targeted to improve this (see Table 5):

- Clinicians use of jargon, the lack of consistency between clinicians and an individual clinician's skill in communication have been previously identified as important factors in patient comprehension and adherence [29, 30] and in the current study all of these aspects impacted reported parental satisfaction and understanding of the conversation. Individual communication skills vary, with one participant noting that some clinicians are 'just better' at it than others. Though this may be an innate strength or weakness within an individual, clinicians need to reflect and improve upon their own communication skills, in the same way they would for any technical or procedural skills required of them. Observation of exemplar mentors and simulation training in communication including discussions with parents of children who have undergone cardiac surgery are useful strategies to improve these skills and gain parent centred perspectives. [26]

- We know that the amount of information given by clinicians can have both positive and negative effects on parental anxiety. [7, 31] This was also reflected in our findings, suggesting that information given to parents needs to be tailored to the information needs of the individual receiving it. Taking the time to know what is right for the parent a clinician is speaking to is an important investment not only in the parent's

https://mc.manuscriptcentral.com/bmjpo

Confidential: For Review Only

understanding, but also in their ability to make decisions and cope with their child's post-operative course. In addition, parents want to know what is likely to happen to *their* child, such that the risk of morbidities would ideally be tailored to the individual characteristics of the child for whom surgery is being discussed. However, as the population of cardiac children become increasingly complex, and as surgical and medical techniques evolve, tailoring assessments of risk is difficult and always subject to some uncertainty. How clinicians support parents and families in coping with uncertainty needs to be an important part of specialist care.

- Stress and time pressure are barriers to informed consent. [32, 33] Participants commented on the difficulties they faced with consent the night before surgery. Where possible, a staged consent approach [34] should be used, such that an initial consultation will be used just to relay information, followed by a period of time to allow consideration by the parents, before a second conversation where consent is actually sought.

Many of our participants commented on the feeling of being 'unprepared'. We suggest future work should seek to understand if clinicians are able to mitigate some of the short and long-term distress experienced by families in this situation, through better communication and understanding of the parental perspective of morbidities and their impact on the child and family.

Confidential: For Review Only

Parents described the importance of other sources of information that they could access whenever they wanted to, such as written information and internet resources. Written information has been found to result in significant decreases in parental anxiety and improved parental comprehension and satisfaction prior to their child's surgery.[35] Multi-media based health information has also been found to reduce parental anxiety [36] although evidence supporting the greater effectiveness of any one method of information provision is lacking, [36, 37] highlighting the importance of individually tailoring information provision. Parents in our study also valued the support offered by staff and in particular the support of other parents who had been through similar experiences, replicating findings with other illness groups.[38] Ensuring the availability of health professionals such as psychologists and medical social workers to provide ongoing support to families could help alleviate parental stress and provide an opportunity for information to be repeated and further discussed.

In this study, the online forum gave us access to many more parents than the focus groups (72 online, 13 at focus groups). This correlates with other reports suggesting the relative ease of online discussion compared to attending in person focus groups. [11, 12, 21]

There are a number of limitations which need to be taken into account when interpreting the findings. Firstly, the data we obtained relied on participants' retrospective recall of conversations (with its inherent limitations on accuracy). Future work to look at clinician – parent communication in real time would provide a valuable insight into what is *actually* said, and the interpretation at the time - not only of parents, but also of clinicians – i.e. what parents understood from the conversation and what clinicians believe the parents understood from the

https://mc.manuscriptcentral.com/bmjpo

Confidential: For Review Only

conversation. Furthermore, using an online forum to collect parent perceptions does not result in the same depth of information that would be possible in individual interviews and the approach precludes probing for further detail.

We recognise that both the online forum and the focus groups can in themselves exclude certain individuals due to lack of familiarity, language barriers, literacy barriers and lack of internet resources. In addition, our study lacked the views of males and non-Caucasians, who were under-represented in our participant sample, despite the chosen locations for the focus groups having a broad ethnic and socio-economic mix. The challenges of including ethnically, culturally and linguistically diverse populations, as well as fathers, in paediatric research studies are well documented. [39, 40] In order to capture these important often unheard views, we must find and adopt an innovative approach that successfully includes minority groups to ensure the broadest capture of parental/family views. Despite specifically choosing a data collection method to increase the accessibility of the research to potential participants, the fact that our participants did not reflect a broad range of ethnic groups or gender also limits the transferability of our findings to the wider population of parents of children with congenital heart disease in the UK.

Finally, we did not collect the same demographic information from participants in the online forum and the focus groups, which limits our ability to describe certain aspects of our participants across both data collection approaches.

Confidential: For Review Only

Conclusions and suggestions for future practice

Our findings indicate the need for an individualised approach to communication about morbidity associated with paediatric cardiac surgery , based on 'high quality conversations', in which certain *key principles* of good communication are followed, as discussed above. This is not unique to paediatric cardiac surgery – rather it is relevant in any situation in which an individual is undergoing a medical or surgical intervention. Future work to investigate the impact of 'good communication' on the short- and long-term psychological morbidity of parents and families would be a valuable next step, together with identifying how this may impact on physical and psychological outcomes in children undergoing cardiac surgery.

Confidential: For Review Only

Funding details

This project was funded by the National Institute for Health Research Health Services and Delivery Research programme (Project No: 12/5005/06).

Acknowledgement

We would like to thank the Children's Heart Federation and Ms. Rehana Ahmed for their assistance in moderating the online forum and focus groups.

Research at Great Ormond Street Hospital is supported by the Great Ormond Street Hospital National Institute of Health Research Biomedical Research Centre.

Contributor statement

K Brown, C Pagel and J Wray planned the study. The forum and focus groups were conducted by R Ahmed and the Children's Heart Federation. V Rajagopal, C Pagel and J Wray analysed and reported results. V Rajagopal wrote the first draft of the manuscript and all authors approved the final version.

Confidential: For Review Only

What is already known about this topic:

- Stress, time pressure and ‘quality’ of communication can adversely affect parental understanding, potentially limiting their ability to make informed decisions

What this study adds:

- A number of targets for improvement in communication have been identified, based on nine suggested principles of ‘good communication’. These principles are:
  - Openness
  - Avoid jargon
  - Include emotional and developmental complications during consent
  - Consistent communication within and across all teams
  - Appropriate timing of conversations
  - Tailor conversations to the individual in front of you
  - Individualise risk – what is likely for *this* particular child?
  - Provide written and audio-visual material
  - Provide a liaison link professional with contacts to parental support groups

Confidential: For Review Only

References

1. Brown KL, Crowe S, Franklin R, McLean A, Cunningham D, Barron D, et al. Trends in 30-day mortality rate and case mix for paediatric cardiac surgery in the UK between 2000 and 2010. Open Heart. 2015;2(1):e000157. Epub 2015/04/22. doi: 10.1136/openhrt-2014-000157. PubMed PMID: 25893099; PubMed Central PMCID: PMCPMC4395835.
2. National Congential Heart Disease Annual Report 2013-2016. Available from: https://www.ucl.ac.uk/nicor/audits/congenital/documents/annual-reports/National_Congenital_Heart_Disease_2013_16_annual_report.
3. NIHR Evaluation TaS. [11 June 2018]. Available from: http://www.nets.nihr.ac.uk/projects/hsdr/12500506.
4. Brown KL, Ridout D, Pagel C, Wray J, Anderson D, Barron DJ, et al. Incidence and risk factors for important early morbidities associated with pediatric cardiac surgery in a UK population. J Thorac Cardiovasc Surg. 2019;158(4):1185-96 e7. Epub 2019/07/30. doi: 10.1016/j.jtcvs.2019.03.139. PubMed PMID: 31353100.
5. Levi RB, Marsick R, Drotar D, Kodish ED. Diagnosis, disclosure, and informed consent: learning from parents of children with cancer. J Pediatr Hematol Oncol. 2000;22(1):3-12. Epub 2000/03/01. doi: 10.1097/00043426-200001000-00002. PubMed PMID: 10695815.
6. Sloper P. Predictors of distress in parents of children with cancer: a prospective study. J Pediatr Psychol. 2000;25(2):79-91. Epub 2000/05/23. doi: 10.1093/jpepsy/25.2.79. PubMed PMID: 10820946.
7. Miller VA, Drotar D, Burant C, Kodish E. Clinician-parent communication during informed consent for pediatric leukemia trials. J Pediatr Psychol. 2005;30(3):219-29. Epub 2005/03/24. doi: 10.1093/jpepsy/jsi032. PubMed PMID: 15784918.
8. Meert KL, Eggly S, Pollack M, Anand KJ, Zimmerman J, Carcillo J, et al. Parents' perspectives on physician-parent communication near the time of a child's death in the pediatric intensive care unit. Pediatr Crit Care Med. 2008;9(1):2-7. Epub 2008/05/15. doi: 10.1097/01.PCC.0000298644.13882.88. PubMed PMID: 18477906; PubMed Central PMCID: PMCPMC3198033.
9. Pagel C, Brown KL, McLeod I, Jepps H, Wray J, Chigaru L, et al. Selection by a panel of clinicians and family representatives of important early morbidities associated with paediatric cardiac surgery suitable for routine monitoring using the nominal group technique and a robust voting process. BMJ Open. 2017;7(5):e014743. Epub 2017/05/31. doi: 10.1136/bmjopen-2016-014743. PubMed PMID: 28554921; PubMed Central PMCID: PMCPMC5729972.
10. Brown KL, Pagel C, Brimmell R, Bull K, Davis P, Franklin RC, et al. Definition of important early morbidities related to paediatric cardiac surgery. Cardiol Young. 2017;27(4):747-56. Epub 2016/09/30. doi: 10.1017/S1047951116001256. PubMed PMID: 27680207.
11. De Simoni A, Shanks A, Mant J, Skelton JR. Making sense of patients' internet forums: a systematic method using discourse analysis. Br J Gen Pract. 2014;64(620):e178-80. Epub 2014/02/26. doi: 10.3399/bjgp14X677671. PubMed PMID: 24567657; PubMed Central PMCID: PMCPMC3933853.
12. Balasooriya-Smeekens C, Bateman A, Mant J, De Simoni A. Barriers and facilitators to staying in work after stroke: insight from an online forum. BMJ Open. 2016;6(4):e009974. Epub 2016/04/08. doi: 10.1136/bmjopen-2015-009974. PubMed PMID: 27053267; PubMed Central PMCID: PMCPMC4823433.
13. Im EO, Chee W. An online forum as a qualitative research method: practical issues. Nurs Res. 2006;55(4):267-73. Epub 2006/07/20. PubMed PMID: 16849979; PubMed Central PMCID: PMCPMC2491331.
14. Im EO, Chee W. Practical guidelines for qualitative research using online forums. Comput Inform Nurs. 2012;30(11):604-11. Epub 2012/08/25. doi: 10.1097/NXN.0b013e318266cade. PubMed PMID: 22918135; PubMed Central PMCID: PMCPMC3727223.
15. Alinia H, Moradi Tuchayi S, Farhangian ME, Huang KE, Taylor SL, Kuo S, et al. Rosacea patients seeking advice: Qualitative analysis of patients' posts on a rosacea support forum. J Dermatolog

Confidential: For Review Only

Treat. 2016;27(2):99-102. Epub 2016/01/28. doi: 10.3109/09546634.2015.1133881. PubMed PMID: 26815357.
16. Jamison J, Sutton S, Mant J, De Simoni A. Online stroke forum as source of data for qualitative research: insights from a comparison with patients' interviews. BMJ Open. 2018;8(3):e020133. Epub 2018/04/01. doi: 10.1136/bmjopen-2017-020133. PubMed PMID: 29602848; PubMed Central PMCID: PMCPMC5884329.
17. Kaufman S, Whitehead KA. Producing, ratifying, and resisting support in an online support forum. Health (London). 2018;22(3):223-39. Epub 2016/02/07. doi: 10.1177/1363459315628043. PubMed PMID: 26851264.
18. Malik S, Coulson NS. The therapeutic potential of the internet: exploring self-help processes in an internet forum for young people with inflammatory bowel disease. Gastroenterol Nurs. 2011;34(6):439-48. Epub 2011/12/02. doi: 10.1097/SGA.0b013e318237a9ba. PubMed PMID: 22129797.
19. McKechnie V, Barker C, Stott J. The effectiveness of an Internet support forum for carers of people with dementia: a pre-post cohort study. J Med Internet Res. 2014;16(2):e68. Epub 2014/03/04. doi: 10.2196/jmir.3166. PubMed PMID: 24583789; PubMed Central PMCID: PMCPMC3961748.
20. Zhao J, Abrahamson K, Anderson JG, Ha S, Widdows R. Trust, empathy, social identity, and contribution of knowledge within patient online communities. Behaviour & Information Technology. 2013;32:1041-8. doi: 10.1080/0144929X.2013.819529.
21. Rhodes SD, Bowie DA, Hergenrather KC. Collecting behavioural data using the world wide web: considerations for researchers. J Epidemiol Community Health. 2003;57(1):68-73. Epub 2002/12/20. PubMed PMID: 12490652; PubMed Central PMCID: PMCPMC1732282.
22. Wray J, Brown K, Tregay J, Crowe S, Knowles R, Bull K, et al. Parents' Experiences of Caring for Their Child at the Time of Discharge After Cardiac Surgery and During the Postdischarge Period: Qualitative Study Using an Online Forum. J Med Internet Res. 2018;20(5):e155. Epub 2018/05/11. doi: 10.2196/jmir.9104. PubMed PMID: 29743157; PubMed Central PMCID: PMCPMC5966654.
23. Available from: http://www.chfed.org.uk/parents-wanted-for-a-study-looking-into-complications-following-heart-surgery/.
24. Coffey A, Atkinson P. Making Sense of Qualitative Data:Complementary Research Strategies. Thousand Oaks, CA: Sage Publications; 1996.
25. Braun V, Clarke V. Using thematic analysis in psychology. Qualitative Research in Psychology. 2006;3:77-101.
26. Levetown M, American Academy of Pediatrics Committee on B. Communicating with children and families: from everyday interactions to skill in conveying distressing information. Pediatrics. 2008;121(5):e1441-60. Epub 2008/05/03. doi: 10.1542/peds.2008-0565. PubMed PMID: 18450887.
27. Scrimin S, Haynes M, Altoe G, Bornstein MH, Axia G. Anxiety and stress in mothers and fathers in the 24 h after their child's surgery. Child Care Health Dev. 2009;35(2):227-33. Epub 2009/02/21. doi: 10.1111/j.1365-2214.2008.00920.x. PubMed PMID: 19228156; PubMed Central PMCID: PMCPMC3496245.
28. Pagel C, Bull C, Utley M, Wray J, Barron DJ, Stoica S, et al. Exploring communication between parents and clinical teams following children's heart surgery: a survey in the UK. BMJ Paediatr Open. 2019;3(1):e000391. Epub 2019/06/18. doi: 10.1136/bmjpo-2018-000391. PubMed PMID: 31206072; PubMed Central PMCID: PMCPMC6542420.
29. Castro CM, Wilson C, Wang F, Schillinger D. Babel babble: physicians' use of unclarified medical jargon with patients. Am J Health Behav. 2007;31 Suppl 1:S85-95. Epub 2007/10/20. doi: 10.5555/ajhb.2007.31.supp.S85. PubMed PMID: 17931142.
30. Zolnierek KB, Dimatteo MR. Physician communication and patient adherence to treatment: a meta-analysis. Med Care. 2009;47(8):826-34. Epub 2009/07/09. doi: 10.1097/MLR.0b013e31819a5acc. PubMed PMID: 19584762; PubMed Central PMCID: PMCPMC2728700.

Confidential: For Review Only

31. Pomicino L, Maccacari E, Buchini S. Levels of anxiety in parents in the 24 hr before and after their child's surgery: A descriptive study. J Clin Nurs. 2018;27(1-2):278-87. Epub 2017/05/26. doi: 10.1111/jocn.13895. PubMed PMID: 28544343.
32. Barnlund DC. The mystification of meaning: doctor-patient encounters. J Med Educ. 1976;51(09):716-25. Epub 1976/09/01. PubMed PMID: 957387.
33. Janis I. Decision making under stress. In: Goldberger L, Breznitz S, editors. Handbook of stress : theoretical and clinical aspects. New York: The Free Press; 1993. p. 56-74.
34. Angiolillo AL, Simon C, Kodish E, Lange B, Noll RB, Ruccione K, et al. Staged informed consent for a randomized clinical trial in childhood leukemia: impact on the consent process. Pediatr Blood Cancer. 2004;42(5):433-7. Epub 2004/03/30. doi: 10.1002/pbc.20010. PubMed PMID: 15049015.
35. Landier M, Villemagne T, Le Touze A, Braik K, Meignan P, Cook AR, et al. The position of a written document in preoperative information for pediatric surgery: A randomized controlled trial on parental anxiety, knowledge, and satisfaction. J Pediatr Surg. 2018;53(3):375-80. Epub 2017/05/01. doi: 10.1016/j.jpedsurg.2017.04.009. PubMed PMID: 28456425.
36. Botngard A, Skranes LP, Skranes J, Dollner H. Multimedia based health information to parents in a pediatric acute ward: a randomized controlled trial. Patient Educ Couns. 2013;93(3):389-93. Epub 2013/05/21. doi: 10.1016/j.pec.2013.04.017. PubMed PMID: 23684375.
37. Franck LS, Spencer C. Informing parents about anaesthesia for children's surgery: a critical literature review. Patient Educ Couns. 2005;59(2):117-25. Epub 2005/11/01. doi: 10.1016/j.pec.2004.11.002. PubMed PMID: 16257615.
38. Shilling V, Morris C, Thompson-Coon J, Ukoumunne O, Rogers M, Logan S. Peer support for parents of children with chronic disabling conditions: a systematic review of quantitative and qualitative studies. Dev Med Child Neurol. 2013;55(7):602-9. Epub 2013/02/21. doi: 10.1111/dmcn.12091. PubMed PMID: 23421818.
39. Costigan CL, Cox MJ. Fathers' participation in family research: is there a self-selection bias? J Fam Psychol. 2001;15(4):706-20. Epub 2002/01/05. PubMed PMID: 11770476.
40. Williams A, Oulton K, Sell D, Wray J. Healthcare professional and interpreter perspectives on working with and caring for non-English speaking families in a tertiary paediatric healthcare setting. Ethn Health. 2017:1-14. Epub 2017/03/10. doi: 10.1080/13557858.2017.1294662. PubMed PMID: 28277020.

Confidential: For Review Only

Table 1: Questions asked on the online forum

1) When thinking about children's heart surgery, what does the word "complication mean to you?
2) When thinking about children's heart surgery, what does the word 'morbidity' mean to you? Can you give an example of how this affected you or your child?
3) As the experience of heart surgery becomes more distant and in the part, and your child is older, are there any difficulties that your child has now which you think may relate to the operation?
4) Thinking about yourself and your family, can you help us understand the impact that any complications had on you, your child or any other family members:<br>➢ While your child was still in hospital?<br>➢ After you got home?
5) We are thinking about measuring and recording how often complications happen, but we want to concentrate on the things that matter most to the child and family. Here is a list of problems we know can happen:<br>• *Children who have heart problems may sometimes experience different types of brain damage and this may lead to disability ranging from loss of hearing – to problems with learning or movement or all types of disability.*<br>• *A child may sometimes need an extra operation during the same stay in hospital that was not planned at the start.*<br>• *Infection may sometimes happen after heart surgery.*<br>• *A child's kidneys may stop working and need to be supported with a machine for a period of time.*<br>• *The muscle that helps a child to breathe may be weakened because the nerve supplying it is bruised or damaged – sometimes an operation is needed to strengthen the breathing muscle (diaphragm muscle).*<br>• *A child may need to stay in intensive care for a long time because he / she needed assistance with breathing or he / she needed tubes to remain in place.*<br>• *A child's gut may not digest milk or food and he / she may need to have nutrition via a drip.*<br>• *A child's heart may become very weak such that a machine to support the body called 'ECMO' was needed.*<br>• *Damage can happen to the nerve supply of the heart so that the child needs to have a pace maker put in to regulate the heart-beat.*<br>➢ Which of these complications seems the worst or most worrying to you?<br>➢ Could you please tell us why you think one or more of these complications is 'worse' than other complications?<br>➢ Are there things that happened to your child after their operation that you think 'went wrong' which should be on this list but isn't?

Confidential: For Review Only

6)	Could you please let us know what information you were provided with regarding the complications of your child's heart surgery?
7)	a) Do you feel you were provided with the right amount of information regarding complications before surgery?<br>b) How much information would you like (more or less)? How much detail is helpful?
8)	What visual (books etc) methods could be developed for letting families know the risk of complications to better inform them about what might happen after surgery?
9)	What do you think about everyone being able to see (on the internet) the numbers of complications occurring after children's heart surgery at different hospitals?
10)	a) Do you have any further comments regarding complications following heart surgery? Is there anything we should know to improve services? Is there anything else you can think of?<br>b) Is there anything we could have done to improve the online study? Do you think we should have asked questions more frequently, for example? Any other suggestions?

Confidential: For Review Only

Table 2: Clinicians' use of language

**Theme: Clinicians' use of language**	
**Sub-themes**	**Quotes**
Comprehension	• *"It's really easy to get yourself tied up in knots with risks and percentages, where they're really not explained properly"*<br>• *"Percentages are rarely accurate anyway, so why do we – as parents – hang on to them so dearly (me included!!!!). We were given 13% but it wasn't properly explained that this was risks (i.e. morbidity) not death alone."*<br>• *"I think it's assumed that parents have a basic understanding of statistics when it's such a complicated measurement and it's not properly explained to us."*<br>• *"It's just jargon…Please…English…so we can understand it!"*<br>• *"…some doctors are better than others…at explaining"*<br>• *"they go into the usual doctor terminology" "they use hypoplastic instead of small, stenotic instead of stiff"*<br>• *"Morbidity to me means death."*<br>• *"Just to talk mum to mum…then you're not getting the medical jargon"*
Consistency	• *"I found a huge discrepancy between the way the cardiologists describe surgery i.e. very optimistic, complications are rare, and the surgeons who spell it out in order to cover themselves. Personally, I prefer the latter as it means when it does happen you are aware of it and know it has happened before, whereas the former makes you feel so unlucky and wondering why things have happened."*<br>• *"We were given very detailed information by the surgeons on the eve of our sons' op but … up until that time we had only seen cardiologists who were really quite blasé… We were told 'he'll be fine' they do switches all the time. Turns out it was far from that…"*

Confidential: For Review Only

Table 3: Being unprepared for complications

**Theme: Being unprepared for complications**	
**Sub-themes**	**Quotes**
Differing priorities of Health Care Professionals (HCP) and families	• *"NG feeding was never something I thought about when we considered the prospect of having a congenital heart disease child."*<br>• *We weren't mentally prepared for the longer stay as we were told 'in and out in 5 days'."*<br>• *"I really wish someone had prepared me for the psychological side effects… anything explaining how trauma and complications can have a negative impact on your child's self-esteem and mental well-being"*<br>• *"Tell parents beforehand… This is very likely going to affect development in growth, height, learning and development and things like that"*<br>• *They do tell you some of the physical things that might happen" but not "how it might affect… a person's behaviour or emotions"*
Timing of consent	• *"You know when they do the consent forms it's usually the night before surgery which… is a bad idea, because you're not taking that in… if you did it a week before… you take in a lot more… it's easy for you to digest and understand"*

Confidential: For Review Only

Table 4: Information needs of families

**Theme: Information needs of families**	
**Sub-themes**	**Quotes**
No right amount of information	• *"Some parents will want to know everything and others want to know as little as possible"*<br>• *"If we knew all the potential outcomes I think signing the consent would have been so much harder"*<br>• *"You can't have a blanket rule of 'We must tell them every possible thing that could go wrong' or, 'We only tell them the most common'. You need to look at it case-by-case"*<br>• *It's such a delicate balance… you've got to try and test the waters with the patient's parents and the patient's themselves to find out how much information… you need to give this person. It has to be an individual case-by-case scenario"*
Types of information	• *"Something to take away and look at and digest in your own time"*<br>• *"Even a simple information sheet… you might not have internet access… you might need to go into your room or sit by the bed and have another look at it a bit later"*<br>• *"Little Hearts Matter… DVD pack for antenatal diagnosis. It was brilliant."*
Access to staff / lay support	• *"Community Liaison Nurses are very useful and parents should be given contact numbers as a matter of course"*<br>• *"knowing who to ask… that there's somebody you can ask about things you might spot… signposting"*

Confidential: For Review Only

Table 5: Principles of good communication

1.	Openness
2.	Avoid jargon
3.	Include emotional and developmental complications during consent
4.	Consistent communication within and across all teams
5.	Appropriate timing of conversations
6.	Tailor conversations to the individual in front of you
7.	Individualise risk – what is likely for *this* particular child?
8.	Provide written and audio-visual material
9.	Provide a liaison link professional with contacts to parental support groups

Confidential: For Review Only

Confidential: For Review Only

Figure 1: Summary of feedback from parents about how complications were communicated to them by healthcare professionals

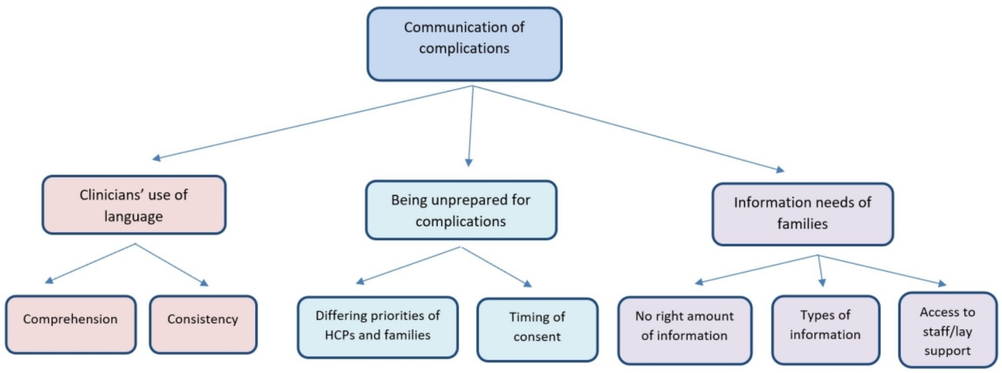

527x240mm (96 x 96 DPI)
